# *BRCA1* Gene as a Potential Marker for Lung Cancer Therapy

**DOI:** 10.3390/ijms27146364

**Published:** 2026-07-17

**Authors:** Matvey M. Tsyganov, Irina A. Tsydenova, Daria S. Dolgasheva, Marina K. Ibragimova

**Affiliations:** 1 Department of Experimental Oncology, Cancer Research Institute, Tomsk National Research Medical Center, Russian Academy of Sciences, 5 Kooperativny Street, 634050 Tomsk, Russia; tsydenova422@gmail.com (I.A.T.); normikus.18.97@gmail.com (D.S.D.); imk1805@yandex.ru (M.K.I.); 2 Biochemistry and Molecular Biology Division, Siberian State Medical University, 2 Moskovsky Trakt, 634050 Tomsk, Russia

**Keywords:** non-small cell lung cancer, *BRCA*-like tumors, *BRCA1*, *BRCA2*, homologous recombination deficiency, survival, chemotherapy efficacy

## Abstract

DNA double-strand breaks (DSBs), caused by various endogenous and exogenous factors, pose a significant threat to genomic stability. Several conserved repair pathways address DSBs, with homologous recombination (HR) being the only mechanism capable of accurately restoring the original DNA sequence. The *BRCA1* gene plays a critical role in HR and is involved in maintaining genomic stability, cell cycle regulation, transcription, and tumor angiogenesis. Germline mutations in *BRCA1* are strongly associated with increased risks of breast, ovarian, and other cancers. Dysfunction of *BRCA1* leads to homologous recombination deficiency (HRD), forcing cells to rely on error-prone repair pathways, which promotes genomic instability and tumorigenesis. Besides hereditary mutations, HRD can also arise in sporadic cancers through epigenetic mechanisms such as promoter hypermethylation and reduced *BRCA1* expression. Although *BRCA1* deficiency is uncommon in lung cancer, *BRCA1* status is considered a potential biomarker for sensitivity to platinum-based chemotherapy and other cytotoxic agents used in lung cancer treatment. However, the impact of *BRCA1* on treatment response and prognosis in lung cancer remains controversial and not fully understood. This review summarizes current evidence on the role of *BRCA1* in modulating chemotherapy response and disease outcomes in lung cancer patients, highlighting its potential as a biomarker for personalized therapy selection. Thus, in this context, the key unresolved issues critical for the development of personalized treatment strategies for lung cancer associated with *BRCA1* alterations include the identification of molecular biomarkers most reliably associated with tumor sensitivity to chemotherapy. In addition, the development of methods for identifying patients with homologous recombination deficiency specifically in lung tumors appears to be of considerable importance, as does a better understanding of how the biological and therapeutic implications of *BRCA1*-related parameters in lung cancer differ from those observed in other tumor types. Addressing these challenges could substantially improve the efficacy of chemotherapy and patient outcomes, while also expanding the opportunities for a personalized approach to treatment selection in patients with lung cancer.

## 1. Introduction

Currently, it has been established that among the numerous constantly arising DNA damages, double-strand breaks (DSBs), which can form as a result of various exogenous or endogenous factors, pose the greatest threat to genomic stability [1]. Several conserved, mechanistically distinct pathways of DSB repair have evolved to address these breaks, including homologous recombination (HR), non-homologous end joining (NHEJ), alternative end joining, and single-strand annealing [1]. It has been shown that the process of homologous recombination is the only DSB repair pathway capable of restoring the original DNA sequence at the site of damage [2,3].

The HR process is facilitated by the functioning of several genes, one of which is *BRCA1*, which encodes a protein composed of 1863 amino acids with multiple functional domains [4]. Earlier studies have indicated that *BRCA1* plays a critical role in maintaining cell stability; DNA repair and recombination [5]; cell cycle regulation [6]; chromatin remodeling [7]; ubiquitination [8]; and the regulation of transcription for various factors, such as p53 [9], transcription factor 1 [10], and c-Myc [11]. Additionally, this gene is significant in regulating the angiogenic process in tumors, which plays a major role in tumor growth and metastasis [12]. *BRCA1* has been identified as a breast cancer (BC) susceptibility gene [13]. Subsequently, it was determined that patients with heterozygous germline mutations in the *BRCA1* (e.g., 5382insC; 2080delA; 300T/G; 3819delGTAAA; 3875del4; 4153delA) or *BRCA2* genes (617delLT, etc.) have an increased risk of developing BC (over 80%), ovarian cancer (40–65%), and other types of cancers [14]. While germline BRCA mutations are major drivers of homologous recombination deficiency (HRD) in hereditary breast and ovarian tumors, the mechanisms underlying HRD in lung cancer are likely to be more heterogeneous and may involve a greater contribution of somatic mutations, chromosomal instability, and epigenetic alterations.

Typically, oncogenesis in carriers of *BRCA1* mutations involves the loss of heterozygosity and the wild-type allele. Cells exhibiting dysfunction in *BRCA1* and *BRCA2* (such as due to the presence of germline mutations) face a deficiency in repairing double-strand breaks through the conservative mechanism of homologous recombination. This deficiency (homologous recombination deficiency, or HRD) leads to DNA damage repair using non-conservative, potentially mutagenic mechanisms, such as non-homologous end joining and single-strand annealing, and also alternative non-homologous end joining (a-NHEJ) (also called microhomology-mediated end joining (MMEJ) or polymerase theta (Polθ)-mediated end joining), which can result in genomic instability and an increased risk of malignancy [15]. Classical non-homologous end joining (c-NHEJ) mediates the direct re-ligation of broken DNA ends and operates throughout the cell cycle, as it does not require a homologous DNA template to complete repair. Alternative non-homologous end joining (a-NHEJ) is likewise active across all phases of the cell cycle and serves as a backup pathway when the major DNA repair mechanisms are compromised. However, a-NHEJ is intrinsically error-prone because it relies on short regions of microhomology, often located at some distance from the double-strand break, to facilitate repair, frequently resulting in deletions and other genomic rearrangements [16]. This contributes to an increased risk of cancer development. However, it’s important to note that current data indicate that, in sporadic forms of cancer, the processes underlying HRD formation can be caused not only by the presence of germline mutations [17] but also by other numerous mechanisms: epigenetic silencing of the wild-type allele through hypermethylation of the *BRCA1* gene promoter [18], hypoexpression [14], etc.

Although *BRCA1* mutations and epigenetic alterations are present in either hereditary or sporadic forms of breast and ovarian tumors, *BRCA1* deficiency due to epigenetic changes or other mechanisms is uncommon in lung tumors [19]. Moreover, *BRCA1* is a molecular marker for a number of cytotoxic agents, such as platinum drugs, as well as drugs involved in depolymerization and disruption of the mitotic spindle, which are used as first-line treatments for patients with lung tumors [20]. Considering that the current standard of care for non-small cell lung cancer (NSCLC) includes treatment with platinum-based chemotherapy [21], this treatment does not benefit many patients, and tumors often develop resistance to therapy.

Nevertheless, information on the impact of *BRCA1* on the treatment and outcome of the disease in patients with lung cancer (LC) is currently considered controversial and not fully understood. It is hypothesized that *BRCA1* and/or *BRCA2* gene-related parameters, including mutations, epigenetic modifications, and gene expression levels, play a key role in determining treatment sensitivity and therapeutic efficacy in non-small cell lung cancer. These alterations contribute to increased genomic instability and the development of pathological mechanisms that reduce the response to conventional therapeutic approaches while simultaneously rendering tumor cells more sensitive to DNA-damaging agents. Furthermore, it can be hypothesized that, across different tumor types and anatomical sites, the development of homologous recombination deficiency may be driven by distinct alterations in homologous recombination repair genes. Thus, this review explores evidence that *BRCA1* gene parameters play a role in modulating the response to chemotherapeutic agents and can be used as a biomarker for selecting therapy in patients with LC, as well as its impact on disease outcome. For the preparation of this literature review, publications indexed in Google Scholar, Scopus, Web of Science, and PubMed and published between 1990 and 2026 were analyzed. The review included studies addressing the main parameters of interest, including non-small cell lung cancer, homologous recombination, BRCAness, somatic and germline mutations, gene expression, hypermethylation, chromosomal instability, patient survival, and treatment efficacy.

## 2. Gene Mutations

To date, the main risk factor for LC is smoking. Other established non-hereditary risk factors also include ionizing radiation, various types of occupational exposure, air pollution, and pulmonary diseases such as tuberculosis and chronic obstructive pulmonary disease [22]. A recent study showed that the incidence rate of lung cancer among people suffering from idiopathic pulmonary fibrosis (IPF) is eight times higher than in the general population [23]. A family history of lung cancer doubles the risk of developing the disease [24]. A number of locus and genes have been identified, in particular 5p15 [25], 6p2 and 15q25.1 [26], *EGFR* [27], *HER2* [28], *BRCA1* [29], *BAP1* [30] and *PARK2* [31], disruptions which may determine a high risk of developing lung cancer.

Analysis of the literature data showed that the frequency of *BRCA1* and *BRCA2* mutations in lung tumors is relatively low (Table 1 and Table 2).

It has been established that the *BRCA1* variant c.139T>C (p.Cys47Arg) was found in a 35-year-old patient diagnosed with lung adenocarcinoma [32]. This mutation has undetermined clinical significance, but it has been shown that another variant of this residue, Cys47Gly, is pathogenic and causes disruption in the homologous recombination process [37]. Huang Y. et al., in their work, conducted a study of the *BRCA1* gene status in tumors from 730 patients with NSCLC. Mutations in the *BRCA1* gene were found in 2.6% of cases. The following mutations were identified: c.2566T>C, c.5066T>C, c.2726del, c.824G>A, c.1418A>G, c.3650C>G, c.4883T>C, c.1945G>T, c.4327C>T, c.571G>A, c.2347A>G, c.2006T>C, and c.211dup. The median overall survival for these patients was 14.0 months [38] (Table S1). The study by Wang L. et al. demonstrated that pathogenic mutations in the *BRCA1*, *BRCA2*, *ERCC6*, *CHEK1*, *MUTYH*, and *RAD51D* genes were prognostic biomarkers of poorer overall survival (OS) in patients with lung cancer compared with patients harboring wild-type variants of these genes [36].

**Table 2:** Mutations of *BRCA2* gene in lung tumor.

Number of Patients	Mutation (Nucleotide Change/Protein Change)	Reference Sequence	Mutation Type	Mutation Class	Frequency, Abs. n., (%)	References
52	c.3450dup (p.Ile1151TyrfsTer7)/I1151F	rs397507668	Frameshift duplication	Pathogenic	1 (1.9)	[39]
	c.3458A>G (p.Lys1153Arg)/K1153R	rs80358594	Missense	CIP	1 (1.9)	
	c.3824T>C (p.Ile1275Thr)/I1275T	rs80358625	Missense	CIP	1 (1.9)	
	c.4421A>G (p.Lys1474Arg)/K1474R	rs780660669	Missense	CIP	1 (1.9)	
	c.5414A>G (p.Asn1805Ser)/N1805S	rs80358765	Missense	CIP	1 (1.9)	
	c.5870T>C (p.Ile1957Thr)/I1957T	rs587782320	Missense	CIP	1 (1.9)	
	c.7095T>A (p.His2365Gln)/H2365Q	rs370708814	Missense	CIP	1 (1.9)	
	c.9155G>A (p.Arg3052Gln)/R3052Q	rs80359171	Missense	CIP	1 (1.9)	
	c.9905G>A (p.Arg3302Lys)/R3302K	rs80359249	Missense	CIP	1 (1.9)	
	c.9925G>A (p.Glu3309Lys)/E3309K	rs80359251	Missense	CIP	1 (1.9)	
11	c.1813dup (p.Ile605AsnfsTer11)/c.1813del (p.Ile605fs)	rs80359306	Frameshift duplication	Pathogenic	1 (9)	[40]
-	c.8350C>T (p.Arg2784Trp)/R2784W	rs80359075	Missense	Pathogenic/Likely pathogenic	-	[32]
22	c.5171T>C (p.Ile1724Thr)	rs80358743	Missense	CIP	1 (4.5)	[41]
15	c.2303_2304del/p.(p.Thr768fs)	-	-	-	1 (6.6)	[42]
126	c.9097del (p.Thr3033fs)/c.9097dup (p.Thr3033fs)	rs397507419	Frameshift deletion	Pathogenic	24 (19)	[43]
148	c.5164_5165del (p.Ser1722fs)/-	rs80359490	Frameshift deletion	Pathogenic	1 (0.7)	[44]
-	c.6952C>T (p.Arg2318Ter)/R2318*	rs80358920	Stop-gain	Pathogenic	1 (9)	[33]
	c.7718T>G (p.Leu2573Ter)/L2573*	rs786203680	Stop-gain	Pathogenic	1 (9)	
	c.5080A>T (p.Arg1694Ter)/R1694*	rs200265692	Stop-gain	Pathogenic	1 (9)	
	c.7805+1G>A /-	rs81002809	Splice donor	Pathogenic	1 (9)	
	c.8951C>G (p.Ser2984Ter)/S2984*	rs80359146	Stop-gain	Pathogenic	1 (9)	
201	c.5593_5594delAT (p.Phe1866TyrfsTer6)/-	-	Frameshift deletion	Pathogenic	1 (0.5)	[34]
	c.5946delT (p.Ser1982ArgfsTer22)/S1982fs	rs80359550	Frameshift deletion	Pathogenic	2 (1)	
1026	c.5163dup (p.Ser1722fs)/p.S1722fs	-	Frameshift duplication	Pathogenic	1 (0.1)	[35]
	-/p.Ile2149fs	-	Frameshift	Pathogenic	1 (0.1)	
	-/p.Lys936fs	-	Frameshift	Pathogenic	1 (0.1)	
	c.1792dup (p.Thr598fs)/p.T598fs	rs886040389	Frameshift duplication	Pathogenic	1 (0.1)	
	c.3109C>T p.(Gln1037Ter)/p.Q1037X	rs80358557	Stop-gain	Pathogenic	1 (0.1)	
	c.3165_3168del (p.Asn1055fs)/p.N1055fs	rs1566227892	Frameshift deletion	Likely pathogenic	1 (0.1)	
63	c.5427C>A (p.Cys1809Ter)/C1809*	rs80359791	Stop-gain	Pathogenic	1 (1.6)	[45]
15	c.71T>G (p.Leu24Ter)/L24*	rs397507902	Stop-gain	Pathogenic	4 (26.7)	[36]
	c.92G>A (p.Trp31Ter)/W31*	rs397508045	Stop-gain	Pathogenic	4 (26.7)	

Note: Pathogenic—pathogenic mutation; CIP—Conflicting interpretations of pathogenicity; *—designation of a “stop codon” in the amino acid name.

For the *BRCA2* gene, the mutation c.8350C>T (p.Arg2784Trp) has been identified, which affects the interaction between *BRCA2* and DSS1 (Table 2) [46]. In a larger cohort of patients with NSCLC, the frequency of somatic mutations in the *BRCA2* gene was shown to be 4.97% (18/362 cases), including mutations such as: c.4951T>C (2 patients), c.4297G>T (2 patients), c.1462A>G (1 patient), c.943T>A (1 patient), T2007S (1 patient), c.5785A>G (1 patient), c.9349C>T (1 patient), c.4109G>T (1 patient), T768S (1 patient), E2260Q (1 patient), R2087K (1 patient), E3167Q (1 patient), S163T (1 patient), T152I (1 patient), E2275Q (1 patient), and S163T (1 patient) [47]. The median overall survival of these patients was 18.0 months.

Importantly, in contrast to breast and ovarian cancers, where germline BRCA1/2 mutations are the predominant mechanism leading to HRD, NSCLC appears to be characterized by a relatively low prevalence of germline *BRCA* alterations and a comparatively greater contribution of somatic mutations to the development of the BRCAness phenotype. In a study by X. Hu et al., an analysis of mutations in the *BRCA1* and *BRCA2* genes was performed. Out of 6220 examined patients with NSCLC, 64 pathogenic mutations and 699 *BRCA* variants of unknown significance were found. Overall, the frequency of the identified mutations was 1.03% (64/6220 cases), with a predominance of mutations in the *BRCA2* gene (49/64, 76.5%). A positive association was established between the presence of mutations in the studied genes and the risk of developing NSCLC [33], while patients in the age cohort under 50 years with mutations were more prone to developing NSCLC (*p* = 0.036). In addition, the authors showed that a number of patients simultaneously revealed the presence of germline and somatic mutations in the *BRCA1/2* genes, which suggests that a somatic *BRCA* mutation may act as a second hit for the development of the tumor process. The following germline mutations were identified: *BRCA2* c.6952C>T (p.Arg2318×); *BRCA2* c.7718T>G (p.Leu2573×); *BRCA2* c.5080A>T (p.Arg1694×); *BRCA2* c.7805+1G>A; *BRCA1* c.2143_2155delinsTCTTT (p.Thr715fs); *BRCA2* c.8951C>G (p.Ser2984×) and somatic mutations: *BRCA2* c.1688G>A (p.Trp563×); *BRCA2* c.9065G>C (p.Arg3022Thr); *BRCA2* c.5082A>G (p.Arg1694=); *BRCA2* c.9299T>A (p.Leu3100×); *BRCA2* c.8026A>T (p.Met2676Leu); *BRCA2* c.8951C>T (p.Ser2984Leu). In conclusion, the authors showed that the use of platinum-based chemotherapy, in the case of germline mutations in patients with NSCLC, shows the greatest effectiveness [33]. Specifically, the authors reported favorable outcomes with platinum-based chemotherapy in previously untreated patients harboring *BRCA* mutations, with median progression-free survival and overall survival of 9.9 and 26 months, respectively.

A study among 257 patients with lung cancer showed the presence of germline *BRCA1* mutations in five patients (c.68_69delAG—in 2 patients; c.5266dupC—in 2 patients; c.224-227delAAAG—in one), *BRCA2* in eight (6175delT—in seven, c.7007 G>C—in one), and *PALB2* (c.917A>T) mutations in one patient. The overall frequency of mutations was 5% (13/257 cases) [48]. Of the eight patients who received platinum compounds, seven responded, but in two cases, progression-free survival was less than 6 months. Additionally, HRD was assessed in three patients [49], and 2/3 of patients (66%) had high levels of homologous recombination deficiency. These patients received platinum-based chemotherapy, and the duration of response to treatment was 23 and 61 months. Another study showed that the frequency of germline and somatic mutations in the *BRCA1* and *BRCA2* genes in patients with NSCLC is 4.9% (459 out of 9324 cases) [44]. The prevalence of somatic mutations in these genes was approximately equal (145 patients (1.56%) versus 169 patients (1.81%), *p* = 0.19). Germline mutations were statistically significantly more common in the *BRCA2* gene (148 cases (1.59%)) than in *BRCA1* (20 cases (0.21%), *p* < 0.0001).

In another study, Remon J. et al. in 2020 [50] analyzed the molecular profile of tumor samples from patients with NSCLC within the Unicancer SAFIR02-Lung/IFCT1301 study (NCT02117167). *BRCA1* and *BRCA2* gene mutations were observed in 20/379 patients. In this case, 8 patients had pathogenetically significant BRCA1/2 mutations: 6 patients had somatic mutations (2 *BRCA1* mutations (c.1069A>T and c.2359G>T) and 4 *BRCA2* mutations (c.2818C>T; c.3317del; c.5993_5994del; c.6800C>G)) and 2 patients had germline *BRCA2* mutations (c.6468_6469del and c.5851_5854del). In this case, the overall response to chemotherapy was 13%. *BRCA1/2* variants of unknown clinical significance were found in 12 patients (3.2%), and the overall response rate to chemotherapy in these patients was 8.3%. A third of tumors carrying pathogenic *BRCA* mutations or variants of unknown significance had biallelic inactivation and a high HRD score. The overall survival of this cohort was 12.8 months, which is consistent with the median overall survival of patients with advanced NSCLC treated with platinum-based chemotherapy. According to the authors of the article, this may indicate the absence of high sensitivity to platinum drugs in patients with *BRCA1/2* mutations in NSCLC [50]. But at the same time, it has been shown that patients with pathogenic mutations in the tumor, at the level of a pronounced trend, have shorter overall survival compared with the group of patients in whom mutations with unidentified clinical significance were found (11.1 versus 17.9 months, log-rank test *p* = 0.07) [50].

In addition, *BRCA1/2* mutational signatures associated with HRD have also shown increased sensitivity to PARP inhibitors in lung cancer cell lines. As a result, PARP inhibitor therapy may be beneficial in cases of LC. In a recently conducted study, mutation data from over 1900 samples were analyzed in non-small cell and small cell lung cancer cells. The results showed the presence of HR gene mutations in 30% of all lung cancer cases. These data suggest that HR genes are frequently mutated in lung cancer, and a personalized approach to treatment is relevant in clinical settings [51]. In 2021, a clinical case of a young patient with no burdened history, having stage IVB metastatic lung adenocarcinoma, was presented [52]. As a result of the analysis, a pathogenic mutation in the *BRCA2* gene was identified in the patient: c.1490C>G (p.Ser497Ter), the presence of which is associated with the risk of developing BC [53]. This shows a rare case of a patient with lung cancer and a germline *BRCA2* mutation who received treatment with immunotherapy and olaparib. Hao J.S.J. et al. also demonstrated the clinical effectiveness of olaparib in a patient with a metastatic form of NSCLC having a *BRCA2* mutation variant c.1411G>T (p.Glu471Ter). Progression-free survival was 8 months with «Olaparib» and was higher than with pembrolizumab and pemetrexed-carboplatin [54].

Another case showed that a patient with lung adenocarcinoma with a *BRCA2* mutation (c.6816_6820del AAGAG, p.G2274Afs×17) had a positive effect from the use of «Olaparib» in combination with gefitinib and recombinant human endostatin with relatively long survival after progression [55]. A similar case was presented for the *BRCA2* mutation c.8401_8403delinsAAAA (p.Phe2801LysfsTer11). The use of olaparib in metastatic lung adenocarcinoma made it possible to achieve partial tumor regression and prolong progression-free survival of the patient [56]. A patient has been described who was diagnosed with concomitant accessory lobular BC and primary lung cancer, both of which were positive for the somatic *BRCA2* mutation (E1593D). An almost complete response to platinum-based chemoradiotherapy was obtained for NSCLC, but BC progression was observed [57]. L. Wang et al. also conducted a study of the occurrence of mutations in the main DNA repair genes. It was found that 7/15 patients had two pathogenic *BRCA1* mutations: c.5095C>T (p.Arg1699Trp) and c.5074G>C (p.Asp1692His) and that 8/15 patients had two *BRCA2* mutations: c.71T>G (p.Leu24Ter) and c.92G>A (p.Trp31Ter) (Table 1 and Table 2), [36]. It is important to note that in both cases, overall survival rates are higher in patients without these mutation variants (with *p* = 0.025 and *p* = 0.059, respectively, for *BRCA1* and *BRCA2*).

## 3. Gene Expression

Unlike breast cancer, where *BRCA* testing is primarily used to identify hereditary predisposition and guide PARP inhibitor therapy, *BRCA1* expression in NSCLC has mainly been investigated as a predictive biomarker of sensitivity to platinum-based chemotherapy and taxanes. In one of the first studies, Taron M. et al. demonstrated the potential role of *BRCA1* mRNA expression in determining the sensitivity of tumors to chemotherapy in NSCLC [58]. Among patients receiving neoadjuvant chemotherapy with gemcitabine/cisplatin, treatment outcomes correlated with *BRCA1* expression levels. Lower *BRCA1* expression (less than 0.61) was associated with better outcomes, while higher expression (greater than 2.45) was associated with worse outcomes. In this case, the median survival was not reached for 15 patients from the group with the lower quartile, whereas for 28 patients with a *BRCA1* level of 0.61–2.45, it was 37.8 months (95% CI, 10.6–65), and for 12 patients with a level greater than 2.45, it was 12.7 months (95% CI 0.28–28.8, *p* = 0.01). In addition, it was found that patients with low expression had a reduced risk of death (HR = 0.206; 95% CI, 0.05–0.83; *p* = 0.026) compared with the group of patients with high *BRCA1* levels.

Following this work, a series of studies began to assess the impact of *BRCA1* expression on the effect of chemotherapy. Rosell R. et al. conducted a phase II clinical prospective study in which a personalized chemotherapy regimen for patients with metastatic NSCLC was determined by the level of *BRCA1* expression. Patients with low *BRCA1* levels (expression less than 4.28) received a gemcitabine/cisplatin chemotherapy regimen, with an intermediate level (expression level 4.28–11.2)—only cisplatin/docetaxel—and with high expression (expression greater than 11.2)—docetaxel. The median progression-free survival for the first group (n = 38) was 11 months, 9 months for 40 patients with an intermediate level of *BRCA1*, and 11 months for 33 patients with a high level of *BRCA1* (*p* = 0.01). Two-year survival was 41.2%, 15.6% and 0%, respectively. As a result, it was found that the appointment of chemotherapy depending on the level of *BRCA1* expression is associated with a high median and two-year survival for some groups of patients with NSCLC [59].

A meta-analysis conducted in 2013 also showed a significant association between *BRCA1* expression and clinical outcome in patients with NSCLC. Low or negative *BRCA1* expression is associated with a higher objective response rate and higher overall survival rates [60]. In particular, according to the results of 17 studies, it was found that in the treatment of patients with NSCLC with platinum-based chemotherapy, a low and/or negative level of *BRCA1* expression indicated a better objective response rate to treatment (OR = 1.70, 95% CI = 1.32–2.18), and longer overall survival and progression-free survival (HR = 1.58, 95% CI = 1.27–1.97 and HR = 1.60, 95% CI = 1.07–2.39 for OS and PFS, respectively). In 4 studies in patients with a taxane-based chemotherapy regimen, a high level of *BRCA1* determined the presence of an objective response to chemotherapy (OR = 0.41, 95% CI = 0.26–0.64). It is important to note that this result is consistent with the results of studies on BC. Reducing the expression of the *BRCA1* gene in BC cell lines increases sensitivity to cisplatin but leads to resistance to paclitaxel and vinorelbine through a defective apoptotic response to these drugs, whereas the opposite phenomenon is observed with normal or high levels of *BRCA1* [61]. This can be explained by the fact that induction of *BRCA1* gene expression after exposure to paclitaxel leads to activation of the mitotic checkpoint and subsequent cell death [6,62]. In this case, a deficiency of the *BRCA1* gene product, on the contrary, leads to the fact that apoptosis of tumor cells under the action of taxanes is not induced.

Unfortunately, despite the significant findings reported by Taron M. et al. in 2004 in a retrospective cohort [58] and the subsequent phase II prospective study conducted by Rosell R. et al. in 2009 [59], the later phase III randomized trial (NCT00617656/GECP-BREC) failed to demonstrate an association between *BRCA1* expression levels and treatment efficacy in patients with NSCLC. This study, which compared the effectiveness of standard chemotherapy with treatment based on the assessment of *BRCA1* expression levels, was prematurely closed because an interim analysis showed a negative effect in terms of progression-free survival in patients in the experimental group (HR, 1.35; *p* = 0.03) [63,64]. However, our studies have shown that a low level of expression of some chemosensitivity genes, in particular *ERCC1*, *BRCA1*, *GSTP1*, is associated with higher rates of metastasis-free survival (log-rank test *p* = 0.0004, *p* = 0.01, *p* = 0.01, respectively) [20]. The 5-year survival rates of patients with high *BRCA1* expression (more than 1) were 55% versus 83% with low expression [20]. Our most recent study allowed us to develop an algorithm for making decisions about prescribing a personalized adjuvant chemotherapy regimen in patients with lung cancer. In particular, the level of *BRCA1* expression was used to prescribe platinum drugs (carboplatin or cisplatin). Thus, the average value of metastasis-free survival rates was 46.2 ± 3.9 months in the group with personalized chemotherapy compared with 22.9 ± 2.65 months in the historical control group (log-rank test *p* = 0.05). Very good results were shown for overall survival. Thus, patients with an individual selection of a chemotherapy regimen had 96% (58.6 ± 2.9 months) survival, compared with the control group, where the lower limit was 48% (26.9 ± 2.39 months) (log-rank test *p* < 0.0001) [65].

**Figure 1:**
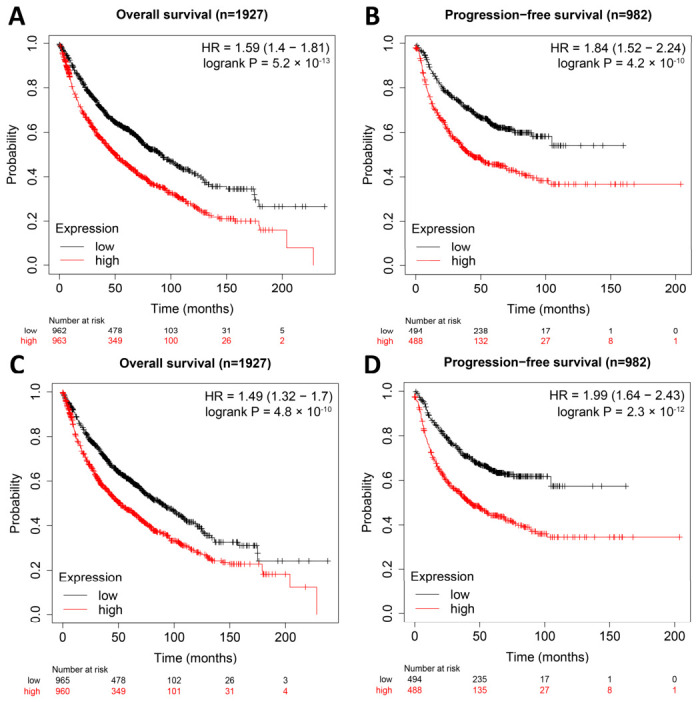
Overall survival (**A**,**C**) and progression-free survival (**B**,**D**) curves for patients with non-small cell lung cancer, based on the expression levels of the *BRCA1* and *BRCA2* genes, respectively.

According to the resource “kmplot.com” which allows assessing the effect of the expression of more than 54 thousand genes on survival in malignant tumors of various locations, it is shown that the level of expression of the *BRCA1* gene in lung tumors (regardless of the stage and histological type of tumor) statistically significantly affects the indicators of overall (Figure 1A) and progression-free survival (Figure 1B). A similar result is shown for the *BRCA2* gene (Figure 1C—overall survival; Figure 1D—progression-free survival).

This result may support the hypothesis that the level of *BRCA1* and *BRCA2* expression has a significant impact on the prognosis of the disease and determines the treatment strategy for patients with NSCLC. Thus, in a study of 100 tumor samples from NSCLC patients, low *BRCA1* expression was found to correlate with a higher response rate to platinum-based chemotherapy. In particular, with a high level of expression, the frequency of complete and partial regressions was only 6% (3/54 cases), while with a low level of *BRCA1*, this figure was 26% (12/46 cases); the differences were statistically significant (*p* = 0.034). Likewise, a low level was associated with longer progression-free survival (*p* = 0.041) and median overall survival (*p* = 0.005) [66]. It is worth paying attention to the fact that in the work of Lee M.N. et al., low or absent expression of *BRCA1* and *BRCA2* was shown in 37% and 34% of the 98 studied lung tumor samples, respectively. Moreover, this was most often found in lung adenocarcinomas (42–44%) [67]. In a recent meta-analysis by Huang Z. et al., it was found that high *BRCA1* expression (relative to low expression) adversely affected the overall survival of patients with NSCLC receiving platinum-based chemotherapy (OR 1.53; 95% CI 1.01–2.31, *p* < 0.05). In this case, no differences were found in the effect of *BRCA1* expression level on event-free survival (OR 1.73; 95% CI 0.98–3.05; *p* > 0.05) [68]. In addition, it is worth noting a combined study of several genes, in particular, the study of the expression of *BRCA1* and the *STMN1*, *MAPT*, and *TUBB3* genes, which play a key role in the regulation of microtubule dynamics [69]. A univariate Cox regression analysis showed that the expression level of *BRCA1* and STMN1 significantly correlated with the 5-year survival of patients with NSCLC (*p* = 0.002 and *p* = 0.006). In patients with high *BRCA1* expression, survival rates were higher. On the contrary, in patients with high *STMN1* expression, survival was lower. *BRCA1* and *STMN1* were independent predictors of prognosis in patients with NSCLC (*p* = 0.008 and *p* = 0.022). A prospective study with an assessment of the expression of the *ERCC1*, *BRCA1*, *RRM1*, and *TUBB3* genes showed that the highest rates of progression-free survival are achieved with the absent expression of the *ERCC1* and *BRCA1* repair genes: 65 and 63% versus 40 and 18% in the group with the presence of expression (*p* = 0.032 and *p* = 0.019). In addition, the authors noted that patients who did not express *ERCC1* and *BRCA1* benefited more from adjuvant cisplatin-based chemotherapy compared with patients who expressed either *ERCC1* or *BRCA1* (HR, 3.102; 95% CI, 1.343–7.163; *p* = 0.008) [70].

In addition, the association between the expression of other homologous recombination genes, including *BARD1*, *BRIP1*, *PARP1*, and *RAD51*, and both overall survival and progression-free survival was evaluated using the kmplot.com resource (Figure S1). The analysis demonstrated a pattern similar to that observed for *BRCA1/2* genes. Specifically, low expression levels of *BRIP1* (HR 1.58; 95% CI 1.36–1.83, *p* = 1.4 × 10^−9^), *PARP1* (HR 1.16; 95% CI 1.03–1.3, *p* = 0.015), and *RAD51* (HR 1.25; 95% CI 1.11–1.41, *p* = 0.00024) were significantly associated with improved overall survival. Moreover, low *RAD51* expression was also significantly associated with improved progression-free survival (HR 1.24; 95% CI 1.01–1.52, *p* = 0.043). Given that these genes are also involved in the core homologous recombination pathway and DNA repair processes, reduced expression may contribute to genomic instability and impaired DNA repair capacity, thereby rendering tumor cells more sensitive to chemotherapy. An interesting finding was observed for *BARD1* (Figure S1A). In contrast to the other genes analyzed, high *BARD1* expression was associated with improved survival outcomes (HR 0.83; 95% CI 0.72–0.96, *p* = 0.013). One possible explanation is that elevated *BARD1* expression may represent a compensatory mechanism for reduced expression of other HR-related genes, thereby contributing to resistance to tumor progression [71]. Thus, despite the central role of *BRCA1/2* genes in the HR pathway, future studies should also consider other genes involved in DNA repair and evaluate them in combination, which may provide a more accurate basis for personalized treatment decision-making.

## 4. Hypermethylation of the *BRCA1/2* Promoter Region

DNA methylation is a covalent biochemical modification resulting in the addition of a methyl group to the 5th carbon position in the pyrimidine ring of cytosine, located in the context of cytosine-phosphate-guanine (CpG) dinucleotides. Hypermethylation often restricts the access of transcription factors to promoters and promotes the binding of methyl-CpG binding domain proteins, which leads to the recruitment of additional proteins associated with suppression and ultimately to the suppression of gene expression [71]. Aberrant DNA methylation is a common phenomenon in tumor tissue, including in NSCLC [72]. Hypermethylation of the promoter region of the *BRCA1* and *BRCA2* repair genes has been demonstrated in sporadic breast and ovarian tumors [18,73]. It has been shown that *BRCA1* promoter methylation levels are higher in platinum-resistant ovarian cancer cell lines and exposure to a demethylating agent sensitized these cells to treatment with these drugs [74]. However, there is no detailed evidence of the potential impact of hypermethylation of the *BRCA1/2* gene promoter region in lung cancer on the effect of treatment and disease prognosis. Hypermethylation of the promoter in the *BRCA1* and *BRCA2* genes in NSCLC tumors has been shown to occur in 30% (29/98 patients) and 42% (41/98 patients), respectively (Table 3). In addition, low mRNA expression was significantly associated with gene hypermethylation (*BRCA1*, *p* < 0.001 and *BRCA2*, *p* < 0.001) [67]. It is important to note that only a single study investigating the promoter methylation status of *BRCA2* was identified. This is particularly intriguing given the relatively large number of studies evaluating *BRCA2* mutations. This discrepancy may likely be explained by the limited number of investigations focusing specifically on *BRCA2* promoter methylation in the context of non-small cell lung cancer (NSCLC). This represents a critically important issue, as the study of epigenetic mechanisms regulating gene activity may reveal either a substantial contribution to, or only a minimal impact on, DNA repair capacity and homologous recombination proficiency. At the same time, a 2011 study showed that the frequency of *BRCA1* methylation in lung tumor tissue is only 10.25% (8/78 patients), compared with normal tissue (2.56%, 2/78 patients), with *p* = 0.098 [75]. Another study showed that *BRCA1* promoter methylation was found in 13 out of 70 patients (18.6%). In this case, recurrence-free survival rates in patients with *BRCA1* methylation were significantly lower (62%) than in patients without *BRCA1* methylation (84%) (*p* = 0.0139) [76]. A similar result was obtained by Weiwei Gao et al.: *BRCA1* promoter hypermethylation was detected in 27/50 (54%), and overall survival was associated with methylation [HR and 95% CI: 2.053 (0.973–6.748)]. The 5-year overall survival rate was only 18% with methylation versus 62% without methylation (*p* = 0.03) [77]. A study of 139 adenocarcinoma lung samples showed a *BRCA1* promoter methylation frequency in 41 patients (29.5%). Interestingly, *EGFR* mutations and aberrant *BRCA1* methylation were mutually exclusive events [78].

It’s important to note that transcription inactivation caused by promoter hypermethylation leads to loss of expression. At the same time, in the case of tumor suppressor genes (e.g., *BRCA1/2*), it seems to be a contradictory event to have a longer survival period in this group of patients. In this case, methylation should be viewed as a continuous and gradual process in which incomplete suppression of gene transcription is possible, and it may lead to active gene expression. Safar A.M. et al. showed that in some suppressor genes, the presence of hypermethylation indicates a protective effect [82]. An analysis of the methylation of four genes, *p16/INK4a, BRCA1*, *RARβ*, and *MGMT*, showed that the absence of hypermethylation in any of the studied genes was associated with shorter survival (HR = 9.3; *p* = 0.001). A multivariate Cox analysis showed that hypermethylation in one or more genes was associated with a better prognosis [79]. The reduction in survival time with increasing methylation confirms the concept that abnormal methylation of the promoter of tumor suppressor genes, including *BRCA1*, is associated with a loss of gene function, which may lead to selective growth advantages of neoplastic cells [79]. However, some studies report the absence of hypermethylation in the promoter regions of repair genes, including *BRCA1* [81].

At the same time, the activation of resistance-associated genes, in addition to hypomethylation of their promoters, may be caused by de novo gene fusions. In the case of BC, tumors with *BRCA1* deficiency, despite the presence of a hypermethylated promoter, have a high level of expression of this gene and its protein product [83]. It has been shown that intragenic deletions of *BRCA1* and loss of *BRCA1* promoter hypermethylation occur, as well as de novo gene fusions, where *BRCA1* expression may be under the transcriptional control of a heterologous promoter [83].

## 5. Chromosomal Instability and Loss of Heterozygosity Regions

In addition, an association between global hypomethylation and chromosomal instability (CIN) has been reported [84]. It has also been suggested that epigenetic changes, such as hypermethylation and hypomethylation, affect certain genomic sites and increase the risk of errors in chromosome segregation, thereby leading to genomic changes and cancer development [85]. Given the high level of chromosomal instability and smoking-associated genomic damage characteristic of lung tumors, the mechanisms leading to HRD in NSCLC may differ substantially from those operating in breast cancer, where hereditary *BRCA* deficiency represents the dominant pathway. To date, the exact mechanisms that integrate genetic aberrations with epigenetic changes are unclear; however, it can be assumed that there are both direct and indirect interactions between genetic damage and changes in methylation patterns. Therefore, profiles linking genetic and epigenetic variations can be used to identify novel clinicopathological markers for the diagnosis and treatment of various cancers. However, there is little specific data on the presence of chromosomal instability in lung tumors. Typically, lung carcinomas have a high frequency of DNA copy number aberrations (CNAs), with gains and losses of entire chromosomes or large regions of chromosomes. These tumors also exhibit simple and complex structural rearrangements responsible for changes in transcription and protein expression, which may include deletions, duplications or amplifications, as well as gene fusions caused by insertions, inversions and translocations [86]. One of the first chromosomal abnormalities in LC was the 3p deletion [87]. The study of large chromosomal abnormalities in LC and their impact on the pathogenesis of the tumor and the effectiveness of treatment is mainly focused on the MYC family genes (*MYCL1*, *MYCN* and *MYC*), members of the EGFR pathways (*EGFR*, *PIK3CA*, *KRAS*), and other genes controlling cell proliferation, such as *FGFR1*, *TP63*, *TERT*, *CCND1*, *CCNE1* and *NKX2-1* [86]. Another study indicates that the most common are amplifications of the 1q21-31, 3q26-qter, 5p13-14 and 8q23-qter regions, where the main oncogenes directly involved in the tumor-forming process are located. Similarly, a decrease in the number of DNA copies (deletions) is most often characteristic in 3p14-21, 8p21-23 and 17p12-13 [88].

In addition, chromosomal instability can be caused by defects in the genes repairing double-strand DNA breaks—*ATM*, *BRCA1* and *BRCA2*, *XRCC5* [67]. Interestingly, the chromosomal regions 2q33-35 and 13q12.3, which include the locus encoding the *XRCC5* and *BRCA2* genes, showed a high frequency of loss of heterozygosity (LOH) sites in NSCLC [89]. In particular, one of the studies found that the frequency of LOH in the *BRCA1* and *BRCA2* genes is 25% and 44%, respectively [67]. Given that *BRCA1/BRCA2* proteins play a key role in p53-dependent elimination of pathological cells, it is not surprising that these proteins are often inactivated or suppressed in NSCLC [90].

## 6. BRCAness in Non-Small Cell Lung Cancer

It has been established that patients with heterozygous germline mutations in the homologous recombination genes *BRCA1* or *BRCA2* (e.g., 5382insC; 2080delA; 300T/G; 3819delGTAAA; 3875del4; 4153delA) or *BRCA2* (617delLT, etc.) have an increased risk of developing BC, ovarian cancer, and other cancers and, more importantly, that germline mutations make the greatest contribution to the formation of homologous recombination deficiency (HRD) [14]. At the same time, current data indicate that, in sporadic forms of cancer, the processes underlying HRD may be due not only to the presence of germline mutations but also to other numerous mechanisms. Tumors phenotypically and genetically similar to familial *BRCA1*-associated BC have similar properties and are defined as “BRCAness,” and these common properties may be important for treatment [15]. To date, the concept of BRCAness defines the pathogenesis and treatment sensitivity of many types of cancer. But to a greater extent, the phenomenon of BRCAness has been shown in BC and ovarian cancer. Thus, in sporadic forms of cancer, *BRCA1* is inactivated by other mechanisms—aberrant methylation of cytosine residues in CpG dinucleotides, low protein and mRNA expression, loss of heterozygosity, etc. It has been shown that the BRCAness phenotype is associated with the most aggressive clinical and pathological features of the tumor, in particular, with a large tumor size (>2 cm, *p* = 0.009), the presence of lymphogenous metastasis (*p* = 0.008), stage 3 disease (*p* = 0.001), high levels of Ki-67 (*p* = 0.001) [91].

**Figure 2:**
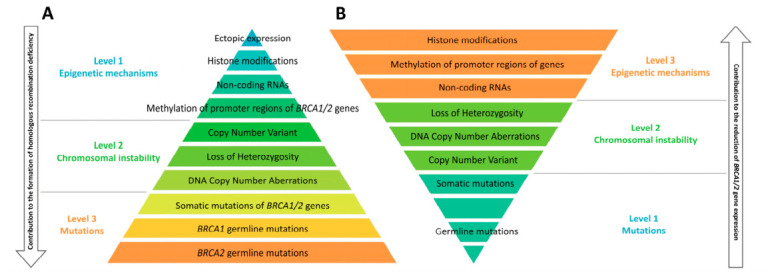
The hypothesized contribution of *BRCA1/2* gene alterations and their regulatory mechanisms to HRD in lung cancer (**A**), and the influence of these factors on gene expression (**B**).

Figure 2 depicts two pyramids. In the first case, it is obvious that the main role in the formation of homologous recombination deficiency is played by germline mutations in the *BRCA1/2* genes. Next, the presence of somatic, usually pathogenic mutations, affects the expression profile of these genes. At the same time, according to the literature data (Table 1 and Table 2), the frequency of mutations in the studied genes is higher than the presence of chromosomal instability. It is important to note that, according to our data, the presence of large chromosomal aberrations (deletions and amplifications) plays an important role not only in the formation of HDR in patients with BC but can also be used to prescribe personalized neoadjuvant chemotherapy [92]. Epigenetic changes make the smallest contribution.

**Figure 3:**
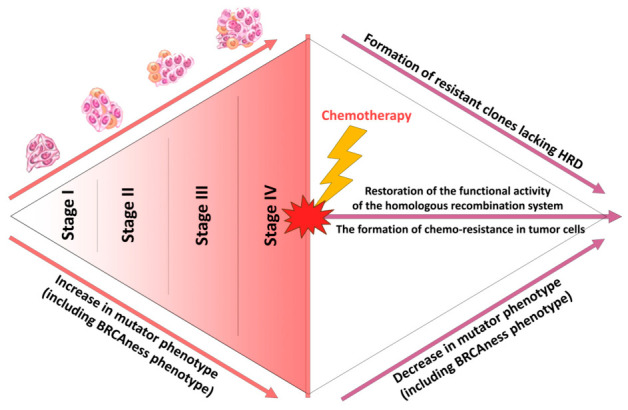
Schematic representation of tumor development and the possible formation of chemoresistance under the influence of chemotherapy.

It is important to note that, despite the fundamental role of germline and somatic mutations in the formation of HRD in patients with LC, the impact on the expression of *BRCA1/2* genes is relatively small due to the action of other mechanisms compensating for *BRCA* deficiency [93]. It should be noted here that the frequency of pathogenic *BRCA1/2* mutations in LC is relatively small, and therefore, one violation is most likely not enough to form HRD (compared with breast cancer or ovarian cancer). If we consider this process within the framework of Knudson’s two-hit hypothesis [94] in addition to the presence of a germline mutation in *BRCA1*, a second hit in the form of chromosomal instability is required to completely impair gene function, which is a characteristic of tumors [95]. It is quite possible that germline mutations affecting the *BRCA1/2* genes, which are involved in maintaining genomic stability, may predispose to a higher probability of CIN and, therefore, to an increased likelihood of loss of heterozygosity [96]. Therefore, studying the formation of HRD in lung tumors, it is necessary to understand that the mechanisms of this phenomenon may be different from those in other locations. In addition, in our opinion, the picture changes completely with respect to HRD during treatment. The initial wide range of violations of homologous recombination mechanisms makes tumor cells increasingly sensitive to DNA-damaging agents, and this ceases to be “beneficial” for the tumor already with direct exposure to chemotherapeutic drugs (Figure 3). In other words, under the influence of therapy, the mechanisms of BRCAness should undergo processes that are the reverse of carcinogenesis and diminish. Due to this, resistant clones devoid of *BRCA* deficiency will be formed, capable of surviving DNA-damaging chemotherapy. It has been established that a possible explanation for the low effectiveness of platinum drugs is that homologous recombination is easily restored under the action of neoadjuvant chemotherapy [15], and promoter demethylation occurs, which confirms our working hypothesis. There is also separate literary evidence for this phenomenon. A study by A.P. Sokolenko [97] found that, despite the high chemosensitivity and rapid reduction of ovarian tumors with germline *BRCA1/2* mutations after neoadjuvant chemotherapy, complete pathological responses to treatment are very rare. The authors showed that this process may be based on a change in the somatic status of *BRCA1* [98]. During NAC, reversion and return of the wild-type allele of the gene occurs, which leads to the restoration of the functional activity of *BRCA1* and the formation of tumor chemoresistance, or under the selective pressure of platinum compounds, the proliferation of already existing clones carrying the wild-type *BRCA1* allele occurs.

It is assumed that chemotherapy has a pronounced mutagenic effect and that its implementation has a strong impact on changing the genetic landscape of the tumor. In this regard, it is important to note that not only the elimination of sensitive clones and the spread and/or replacement of already existing tumor clones carrying the wild-type *BRCA1* allele can occur but also the emergence of new mutational changes in genes, including, for example, amplification of the 17q21.31 locus containing the *BRCA1* gene, even if its allelic deletion was initially in the tumor. And, this may be one of the first mechanisms for restoring the functionality of the homologous recombination process, which leads to the fact that some HRD-positive tumors turn out to be insensitive to DNA-damaging chemotherapy. Our studies on breast cell lines confirmed this hypothesis. It was found that the selective action of cisplatin restored the normal copy number for the *BRCA1*, *CDK12*, *CHEK1* and *RAD51D* genes in the MCF-7 cell line. The appearance of amplifications was revealed for *BRCA2* and *PALB2*. A statistically significant increase in the expression of the *BRCA1* (*p* = 0.04), *BRCA2* (*p* = 0.02), *PALB2* (*p* = 0.01) and *RAD51D* (*p* = 0.05) genes was also shown. A similar result was shown for MDA-MB-231: it was shown that all identified loci with deletions, where the *BRCA2*, *BARD1*, *CHEK2*, *PALB2* and *RAD54L* genes are localized, were restored to normal copy number under the action of cisplatin. The emergence of amplifications was registered for *BRCA1*, *BRIP1*, *FANCL*, *RAD51B*, *PARP1*. A similar result was shown for docetaxel. An increase in the expression level is characteristic of the genes *BRCA1* (*p* = 0.02), *BRCA2* (*p* = 0.02), *CHEK2* (*p* = 0.05), *FANCL* (*p* = 0.04), *PALB2* (*p* = 0.05), *RAD51C* (*p* = 0.02), *PARP1* (*p* = 0.02), which corresponds to the emergence of amplifications. But, in the MDA-MB-468 cell culture, where the initial genetic profile of the homologous recombination genes does not have deletions, an increase in the copy number of only the *BRCA1* gene is observed. The effect of docetaxel does not affect this cell culture at all. The expression level of *BRCA1* increases in direct proportion to the duration of the drug action [99].

The study of the mechanisms and patterns of restoration of the functionality of the homologous recombination process is relevant from the point of view of personalizing the treatment of HRD-positive patients.

BRCAness was investigated not only as a prognostic biomarker but also as a new therapeutic strategy through its pharmacological induction. Interesting results were obtained by Min A. et al. (2015) and Mio C. et al. (2019), who found that the induction of the BRCAness phenotype can be achieved through epigenetic suppression of *BRCA* and enhancement of the activity of chemotherapeutic drugs [100,101].

Thus, to date, there is no clear concept of the formation of HRD in the process of carcinogenesis and its changes in the process of treatment: at what stages of carcinogenesis do certain disorders of the *BRCA* genes appear; which disorders determine sensitivity to platinum drugs and other DNA-damaging agents; and how quickly do they change (their actual “persistence”) under the action of chemotherapeutic drugs, determining the formation of resistance or insensitivity to therapy? All these questions require further investigation.

## 7. Conclusions

Based on the analysis of the available literature, the findings support the hypothesis that various *BRCA1*-related parameters, as well as those associated with *BRCA2*, may contribute to the development of homologous recombination deficiency in non-small cell lung cancer and influence treatment efficacy. Furthermore, the evidence suggests that, in contrast to other tumor types, the BRCAness phenotype in lung cancer is likely to be driven predominantly by somatic and, less frequently, germline mutations. Importantly, although the concept of *BRCA* deficiency originated from studies of breast and ovarian cancers, accumulating evidence suggests that the biological basis, clinical implications, and therapeutic relevance of *BRCA1/2* alterations in NSCLC may be substantially different. In particular, lung cancer appears to exhibit a more heterogeneous spectrum of mechanisms leading to HRD, involving not only *BRCA1/2* mutations but also alterations in other homologous recombination genes, chromosomal instability, and potentially epigenetic mechanisms. Nevertheless, additional large-scale retrospective and prospective studies are required to achieve a more accurate and comprehensive understanding of this issue. In addition, in vitro experiments using lung cancer cell lines are needed to further elucidate the mechanisms by which different *BRCA1*-related alterations affect DNA repair processes and treatment resistance in response to various chemotherapeutic agents.

Thus, the role of *BRCA1/2* genes as drivers of lung cancer mutations is still unclear and necessitates confirmation through epidemiological research. In light of the conflicting evidence, this literature review sought to identify the potential of *BRCA1/2* gene parameters to predict treatment response and prognosis, ultimately facilitating a personalized approach to the management of non-small cell lung cancer patients.

## Figures and Tables

**Table 1:** Mutations of *BRCA1* gene in lung tumor.

Number of Patients	Mutation (Nucleotide Change/Protein Change)	Reference Sequence	Mutation Type	Mutation Class	Frequency, Abs. n., (%)	References
-	c.139A>G (p.Cys47Arg)/C47R	rs80357370	Missense	Pathogenic	-	[32]
-	c.2143_2155delinsTCTTT (p.Thr715SerfsTer8)/T668fs	rs1567796807	Frameshift delins	Pathogenic	-	[33]
201	c.122A>T (p.His41Leu)/H41L	rs80357276	Missense	Pathogenic/Likely pathogenic	1 (0.5)	[34]
	c.4035del (p.Glu1346fs)/E1299fs	rs80357711	Frameshift deletion	Pathogenic	1 (0.5)	
	c.520C>T (p.Gln174Ter)/Q174*	rs1567806048	Stop-gain	Pathogenic	1 (0.5)	
1026	-/p.I1824fs	-	Frameshift	Likely pathogenic	1 (0.1)	[35]
	-/IVS5332+1G>-	-	Missense	Likely pathogenic	1 (0.1)	
15	c.5095C>T (p.Arg1699Trp)/R1699W	rs55770810	Missense	Pathogenic	3 (20.0)	[36]
	c.5074G>C (p.Asp1692His)/D1692H	rs80187739	Missense	Pathogenic	4 (26.7)	

Note: Pathogenic—pathogenic mutation; *—designation of a “stop codon” in the amino acid name.

**Table 3:** *BRCA1* and *BRCA2* promoter methylation frequency in lung cancer patients.

Number of Patients	Frequency, Abs. n., (%)	References
*BRCA1*		
98	29 (30.0)	[67]
78	8 (10.25)	[75]
70	13 (18.6)	[76]
50	27 (54.0)	[77]
139	41 (29.5)	[78]
30	16 (53.3)	[79]
28	18 (64.3)	[80]
158	4 (2.5)	[19]
56	0 (0.0)	[81]
*BRCA2*		
98	41 (42.0)	[67]

## Data Availability

No new data were created or analyzed in this study. Data sharing is not applicable to this article.

## Supplementary Materials

The following supporting information can be downloaded at: https://www.mdpi.com/article/10.3390/ijms27146364/s1. References [29,32,33,34,35,36,39,40,41,42,43,44,45,102] are cited in the supplementary materials.

## References

[1] Tarsounas M., Sung P. (2020). The antitumorigenic roles of BRCA1–BARD1 in DNA repair and replication. Nat. Rev. Mol. Cell Biol..

[2] Scully R., Panday A., Elango R., Willis N.A. (2019). DNA double-strand break repair-pathway choice in somatic mammalian cells. Nat. Rev. Mol. Cell Biol..

[3] Her J., Bunting S.F. (2018). How cells ensure correct repair of DNA double-strand breaks. J. Biol. Chem..

[4] Miki Y., Swensen J., Shattuck-Eidens D., Futreal P.A., Harshman K., Tavtigian S., Liu Q., Cochran C., Bennett L.M., Ding W. (1994). A strong candidate for the breast and ovarian cancer susceptibility gene BRCA1. Science.

[5] Wang Y., Cortez D., Yazdi P., Neff N., Elledge S.J., Qin J. (2000). BASC, a super complex of BRCA1-associated proteins involved in the recognition and repair of aberrant DNA structures. Genes Dev..

[6] Mullan P.B., Quinn J.E., Gilmore P.M., McWilliams S., Andrews H., Gervin C., McCabe N., McKenna S., White P., Song Y.-H. (2001). BRCA1 and GADD45 mediated G2/M cell cycle arrest in response to antimicrotubule agents. Oncogene.

[7] Ye Q., Hu Y.-F., Zhong H., Nye A.C., Belmont A.S., Li R. (2001). BRCA1-induced large-scale chromatin unfolding and allele-specific effects of cancer-predisposing mutations. J. Cell Biol..

[8] Starita L.M., Parvin J.D. (2006). Substrates of the BRCA1-dependent ubiquitin ligase. Cancer Biol. Ther..

[9] Ouchi T., Monteiro A.N., August A., Aaronson S.A., Hanafusa H. (1998). BRCA1 regulates p53-dependent gene expression. Proc. Natl. Acad. Sci. USA.

[10] Houvras Y., Benezra M., Zhang H., Manfredi J.J., Weber B.L., Licht J.D. (2000). BRCA1 physically and functionally interacts with ATF1. J. Biol. Chem..

[11] Murray M., Mullan P., Harkin D. (2007). Role played by BRCA1 in transcriptional regulation in response to therapy. Biochem. Soc. Trans..

[12] Caine G., Blann A., Stonelake P., Ryan P., Lip G. (2003). Plasma angiopoietin-1, angiopoietin-2 and Tie-2 in breast and prostate cancer: A comparison with VEGF and Flt-1. Eur. J. Clin. Investig..

[13] Hall J.M., Lee M.K., Newman B., Morrow J.E., Anderson L.A., Huey B., King M.-C. (1990). Linkage of early-onset familial breast cancer to chromosome 17q21. Science.

[14] Turner N., Tutt A., Ashworth A. (2004). Hallmarks of’BRCAness’ in sporadic cancers. Nat. Rev. Cancer.

[15] Lord C.J., Ashworth A. (2016). BRCAness revisited. Nat. Rev. Cancer.

[16] Saha J., Davis A.J. (2016). Unsolved mystery: The role of BRCA1 in DNA end-joining. J. Radiat. Res..

[17] Futreal P.A., Liu Q., Shattuck-Eidens D., Cochran C., Harshman K., Tavtigian S., Bennett L.M., Haugen-Strano A., Swensen J., Miki Y. (1994). BRCA1 mutations in primary breast and ovarian carcinomas. Science.

[18] Esteller M., Silva J.M., Dominguez G., Bonilla F., Matias-Guiu X., Lerma E., Bussaglia E., Prat J., Harkes I.C., Repasky E.A. (2000). Promoter hypermethylation and BRCA1 inactivation in sporadic breast and ovarian tumors. JNCI J. Natl. Cancer Inst..

[19] Marsit C.J., Liu M., Nelson H.H., Posner M., Suzuki M., Kelsey K.T. (2004). Inactivation of the Fanconi anemia/BRCA pathway in lung and oral cancers: Implications for treatment and survival. Oncogene.

[20] Tsyganov M.M., Rodionov E.O., Pevzner A.M., Ibragimova M.K., Miller S.V., Cheremisina O.V., Frolova I.G., Tuzikov S.A., Litviakov N.V. (2020). Prognostic significance of ERCC1, RRM1, TOP1, TOP2A, TYMS, TUBB3, GSTP1 and BRCA1 mRNA expressions in patients with non-small-cell lung cancer receiving a platinum-based chemotherapy. J. BUON.

[21] Allingham-Hawkins D., Lea A., Levine S. (2010). ERCC1 expression analysis to guide therapy in non-small cell lung cancer. PLoS Curr..

[22] Malhotra J., Malvezzi M., Negri E., La Vecchia C., Boffetta P. (2016). Risk factors for lung cancer worldwide. Eur. Respir. J..

[23] Choi W.-I., Park S.H., Park B.J., Lee C.W. (2018). Interstitial lung disease and lung Cancer development: A 5-year Nationwide population-based study. Cancer Res. Treat..

[24] Coté M.L., Liu M., Bonassi S., Neri M., Schwartz A.G., Christiani D.C., Spitz M.R., Muscat J.E., Rennert G., Aben K.K. (2012). Increased risk of lung cancer in individuals with a family history of the disease: A pooled analysis from the International Lung Cancer Consortium. Eur. J. Cancer.

[25] McKay J.D., Hung R.J., Gaborieau V., Boffetta P., Chabrier A., Byrnes G., Zaridze D., Mukeria A., Szeszenia-Dabrowska N., Lissowska J. (2008). Lung cancer susceptibility locus at 5p15.33. Nat. Genet..

[26] Hung R.J., McKay J.D., Gaborieau V., Boffetta P., Hashibe M., Zaridze D., Mukeria A., Szeszenia-Dabrowska N., Lissowska J., Rudnai P. (2008). A susceptibility locus for lung cancer maps to nicotinic acetylcholine receptor subunit genes on 15q25. Nature.

[27] Yu H.A., Arcila M.E., Fleischut M.H., Stadler Z., Ladanyi M., Berger M.F., Robson M., Riely G.J. (2014). Germline EGFR T790M mutation found in multiple members of a familial cohort. J. Thorac. Oncol..

[28] Yamamoto H., Higasa K., Sakaguchi M., Shien K., Soh J., Ichimura K., Furukawa M., Hashida S., Tsukuda K., Takigawa N. (2014). Novel germline mutation in the transmembrane domain of HER2 in familial lung adenocarcinomas. J. Natl. Cancer Inst..

[29] Wang Y., McKay J.D., Rafnar T., Wang Z., Timofeeva M.N., Broderick P., Zong X., Laplana M., Wei Y., Han Y. (2014). Rare variants of large effect in BRCA2 and CHEK2 affect risk of lung cancer. Nat. Genet..

[30] Abdel-Rahman M.H., Pilarski R., Cebulla C.M., Massengill J.B., Christopher B.N., Boru G., Hovland P., Davidorf F.H. (2011). Germline BAP1 mutation predisposes to uveal melanoma, lung adenocarcinoma, meningioma, and other cancers. J. Med. Genet..

[31] Xiong D., Wang Y., Kupert E., Simpson C., Pinney S.M., Gaba C.R., Mandal D., Schwartz A.G., Yang P., De Andrade M. (2015). A recurrent mutation in PARK2 is associated with familial lung cancer. Am. J. Hum. Genet..

[32] Donner I., Katainen R., Sipilä L.J., Aavikko M., Pukkala E., Aaltonen L.A. (2018). Germline mutations in young non-smoking women with lung adenocarcinoma. Lung Cancer.

[33] Hu X., Yang D., Li Y., Li L., Wang Y., Chen P., Xu S., Pu X., Zhu W., Deng P. (2019). Prevalence and clinical significance of pathogenic germline BRCA1/2 mutations in Chinese non-small cell lung cancer patients. Cancer Biol. Med..

[34] Ricciuti B., Recondo G., Spurr L.F., Li Y.Y., Lamberti G., Venkatraman D., Umeton R., Cherniack A.D., Nishino M., Sholl L.M. (2020). Impact of DNA damage response and repair (DDR) gene mutations on efficacy of PD-(L) 1 immune checkpoint inhibition in non–small cell lung cancer. Clin. Cancer Res..

[35] Liu M., Liu X., Suo P., Gong Y., Qu B., Peng X., Xiao W., Li Y., Chen Y., Zeng Z. (2020). The contribution of hereditary cancer-related germline mutations to lung cancer susceptibility. Transl. Lung Cancer Res..

[36] Wang L., Ma Y., Han W., Yang Q., Jamil M. (2023). Whole exome sequencing reveals clinically important pathogenic mutations in DNA repair genes across lung cancer patients. Am. J. Cancer Res..

[37] Ransburgh D.J., Chiba N., Ishioka C., Toland A.E., Parvin J.D. (2010). Identification of breast tumor mutations in BRCA1 that abolish its function in homologous DNA recombination. Cancer Res..

[38] Huang Y., Xu C., Wang W., Zhang Q., Zhuang W., Zhu Y., Chen G., Fang M., Lv T., Song Y. (2019). EP1. 03-28 Frequency and Molecular Characteristics of BRCA1 Mutations in Non-Small Cell Lung Cancer from East Asian Patients. J. Thorac. Oncol..

[39] Parry E.M., Gable D.L., Stanley S.E., Khalil S.E., Antonescu V., Florea L., Armanios M. (2017). Germline mutations in DNA repair genes in lung adenocarcinoma. J. Thorac. Oncol..

[40] Fan Y., Zhu X., Xu Y., Lu X., Xu Y., Wang M., Xu H., Ding J., Ye X., Fang L. (2018). Cell-cycle and DNA-damage response pathway is involved in leptomeningeal metastasis of non–small cell lung cancer. Clin. Cancer Res..

[41] Pietanza M.C., Waqar S.N., Krug L.M., Dowlati A., Hann C.L., Chiappori A., Owonikoko T.K., Woo K.M., Cardnell R.J., Fujimoto J. (2018). Randomized, double-blind, phase II study of temozolomide in combination with either veliparib or placebo in patients with relapsed-sensitive or refractory small-cell lung cancer. J. Clin. Oncol..

[42] Lu S., Yu Y., Li Z., Yu R., Wu X., Bao H., Ding Y., Shao Y.W., Jian H. (2019). EGFR and ERBB2 germline mutations in Chinese lung cancer patients and their roles in genetic susceptibility to cancer. J. Thorac. Oncol..

[43] Lu J., Zhong H., Wu J., Chu T., Zhang L., Li H., Wang Q., Li R., Zhao Y., Gu A. (2019). Circulating DNA-based sequencing guided anlotinib therapy in non-small cell lung cancer. Adv. Sci..

[44] Fang W., Cai X., Zhou H., Wang Y., Zhang Y., Hong S., Shao Y., Zhang L. (2019). BRCA1/2 germline mutations and response to PARP inhibitor treatment in lung cancer. J. Clin. Oncol..

[45] Wu S., Zhang Y., Zhang Y., Chen L.H., Ouyang H.F., Xu X., Du Y., Ti X.Y. (2023). Mutational landscape of homologous recombination-related genes in small-cell lung cancer. Cancer Med..

[46] Li J., Zou C., Bai Y., Wazer D., Band V., Gao Q. (2006). DSS1 is required for the stability of BRCA2. Oncogene.

[47] Lai J., Xu C., Wang W., Zhang Q., Zhuang W., Zhu Y., Huang Y., Chen Y., Chen G., Fang M. (2018). P1.03-27 somatic mutations in BRCA2 genes are associated with prognosis in chinese non-small-cell lung cancer patients. J. Thorac. Oncol..

[48] Kadouri L., Rottenberg Y., Zick A., Hamburger T., Lipson D., Peretz T., Nechushtan H. (2019). Homologous recombination in lung cancer, germline and somatic mutations, clinical and phenotype characterization. Lung Cancer.

[49] Swisher E.M., Lin K.K., Oza A.M., Scott C.L., Giordano H., Sun J., Konecny G.E., Coleman R.L., Tinker A.V., O’Malley D.M. (2017). Rucaparib in relapsed, platinum-sensitive high-grade ovarian carcinoma (ARIEL2 Part 1): An international, multicentre, open-label, phase 2 trial. Lancet Oncol..

[50] Remon J., Besse B., Leary A., Bièche I., Job B., Lacroix L., Auguste A., Mauduit M., Audigier-Valette C., Raimbourg J. (2020). Somatic and germline BRCA 1 and 2 mutations in advanced NSCLC from the SAFIR02-lung trial. JTO Clin. Res. Rep..

[51] Diossy M., Sztupinszki Z., Borcsok J., Krzystanek M., Tisza V., Spisak S., Rusz O., Timar J., Csabai I., Fillinger J. (2021). A subset of lung cancer cases shows robust signs of homologous recombination deficiency associated genomic mutational signatures. npj Precis. Oncol..

[52] Waddington T., Mambetsariev I., Pharaon R., Fricke J., Baroz A.R., Romo H., Ghanem B., Gray S., Salgia R. (2021). Therapeutic Potential of Olaparib in Combination with Pembrolizumab in a Young Patient with a Maternally Inherited BRCA2 Germline Variant: A Research Report. Clin. Lung Cancer.

[53] Mehrgou A., Akouchekian M. (2016). The importance of BRCA1 and BRCA2 genes mutations in breast cancer development. Med. J. Islam. Repub. Iran.

[54] Hao J.S.J., Hoai C.S., Weng D.T.S., Ngeow J., Chiang J. (2022). Case report: Olaparib use in metastatic lung adenocarcinoma with BRCA2 pathogenic variant. Mol. Case Stud..

[55] Zhang L., Wang J., Cui L.-Z., Wang K., Yuan M.-M., Chen R.-R., Zhang L.-J. (2021). Successful treatment of refractory lung adenocarcinoma harboring a germline BRCA2 mutation with olaparib: A case report. World J. Clin. Cases.

[56] Wu C., Fan M., Hu Y. (2022). Response to olaparib in metastatic lung adenocarcinoma with germline BRCA2 mutation: A case report. Anti-Cancer Drugs.

[57] Cheng Y., Li N., Eapen A., Parajuli R., Mehta R. (2019). Somatic BRCA2 mutation-positive concurrent accessory male breast cancer (BC) and non-small cell lung cancer (NSCLC): Excellent efficacy of palbociclib, fulvestrant and leuprolide in platinum-exposed and endocrine-refractory BC associated with cyclin D1 and FGFR1 amplification and of carboplatin, paclitaxel and radiation in NSCLC. Case Rep. Oncol..

[58] Taron M., Rosell R., Felip E., Mendez P., Souglakos J., Ronco M.S., Queralt C., Majo J., Sanchez J.M., Sanchez J.J. (2004). BRCA1 mRNA expression levels as an indicator of chemoresistance in lung cancer. Hum. Mol. Genet..

[59] Rosell R., Perez-Roca L., Sanchez J.J., Cobo M., Moran T., Chaib I., Provencio M., Domine M., Sala M.A., Jimenez U. (2009). Customized treatment in non-small-cell lung cancer based on EGFR mutations and BRCA1 mRNA expression. PLoS ONE.

[60] Yang Y., Xie Y., Xian L. (2013). Breast cancer susceptibility gene 1 (BRCA1) predict clinical outcome in platinum-and toxal-based chemotherapy in non-small-cell lung cancer (NSCLC) patients: A system review and meta-analysis. J. Exp. Clin. Cancer Res..

[61] Lafarge S., Sylvain V., Ferrara M., Bignon Y.-J. (2001). Inhibition of BRCA1 leads to increased chemoresistance to microtubule-interfering agents, an effect that involves the JNK pathway. Oncogene.

[62] Quinn J.E., Kennedy R.D., Mullan P.B., Gilmore P.M., Carty M., Johnston P.G., Harkin D.P. (2003). BRCA1 functions as a differential modulator of chemotherapy-induced apoptosis. Cancer Res..

[63] Moran T., Cobo M., Domine M., Sanchez-Ronco M., Bover I., Provencio M., Massuti B., Vergnenegre A., Lopez-Vivanco G., Robinet G. (2013). Interim analysis of the Spanish Lung Cancer Group (SLCG) BRCA1-RAP80 Expression Customization (BREC) randomized phase III trial of customized therapy in advanced non-small cell lung cancer (NSCLC) patients (p) (NCT00617656/GECP-BREC). J. Clin. Oncol..

[64] Moran T., Wei J., Cobo M., Qian X., Domine M., Zou Z., Bover I., Wang L., Provencio M., Yu L. (2014). Two biomarker-directed randomized trials in European and Chinese patients with nonsmall-cell lung cancer: The BRCA1-RAP80 Expression Customization (BREC) studies. Ann. Oncol..

[65] Tsyganov M.M., Rodionov E.O., Ibragimova M.K., Miller S.V., Cheremisina O.V., Frolova I.G., Tuzikov S.A., Litviakov N.V. (2022). Personalized Prescription of Chemotherapy Based on Assessment of mRNA Expression of BRCA1, RRM1, ERCC1, TOP1, TOP2α, TUBβ3, TYMS, and GSTP1 Genes in Tumors Compared to Standard Chemotherapy in the Treatment of Non-Small-Cell Lung Cancer. J. Pers. Med..

[66] Papadaki C., Sfakianaki M., Ioannidis G., Lagoudaki E., Trypaki M., Tryfonidis K., Mavroudis D., Stathopoulos E., Georgoulias V., Souglakos J. (2012). ERCC1 and BRAC1 mRNA expression levels in the primary tumor could predict the effectiveness of the second-line cisplatin-based chemotherapy in pretreated patients with metastatic non-small cell lung cancer. J. Thorac. Oncol..

[67] Lee M.-N., Tseng R.-C., Hsu H.-S., Chen J.-Y., Tzao C., Ho W.L., Wang Y.-C. (2007). Epigenetic inactivation of the chromosomal stability control genes BRCA1, BRCA2, and XRCC5 in non–small cell lung cancer. Clin. Cancer Res..

[68] Huang Z., Xiong G. (2022). BRCA1 expression associated with the prognostic value of platinum-based chemotherapy for stage II–IV non-small cell lung cancer: A meta-analysis. Int. J. Biol. Markers.

[69] Wang M., Li W., Xing X., Zhang D., Lei J., Li G. (2017). BRCA1 and STMN1 as prognostic markers in NSCLCs who received cisplatin-based adjuvant chemotherapy. Oncotarget.

[70] Huang Z.L., Cao X., Luo R.Z., Chen Y.F., Zhu L.C., Wen Z. (2016). Analysis of ERCC1, BRCA1, RRM1 and TUBB3 as predictors of prognosis in patients with non-small cell lung cancer who received cisplatin-based adjuvant chemotherapy: A prospective study. Oncol. Lett..

[71] Kondo Y., Shen L., Cheng A.S., Ahmed S., Boumber Y., Charo C., Yamochi T., Urano T., Furukawa K., Kwabi-Addo B. (2008). Gene silencing in cancer by histone H3 lysine 27 trimethylation independent of promoter DNA methylation. Nat. Genet..

[72] Schiffmann I., Greve G., Jung M., Lübbert M. (2016). Epigenetic therapy approaches in non-small cell lung cancer: Update and perspectives. Epigenetics.

[73] Gras E., Cortes J., Diez O., Alonso C., Matias-Guiu X., Baiget M., Prat J. (2001). Loss of heterozygosity on chromosome 13q12–q14, BRCA-2 mutations and lack of BRCA-2 promoter hypermethylation in sporadic epithelial ovarian tumors. Cancer.

[74] Wang Y.Q., Zhang J.R., Li S.D., He Y.Y., Yang Y.X., Liu X.L., Wan X.P. (2010). Aberrant methylation of breast and ovarian cancer susceptibility gene 1 in chemosensitive human ovarian cancer cells does not involve the phosphatidylinositol 3′-kinase–Akt pathway. Cancer Sci..

[75] Zhang Y., Wang R., Song H., Huang G., Yi J., Zheng Y., Wang J., Chen L. (2011). Methylation of multiple genes as a candidate biomarker in non-small cell lung cancer. Cancer Lett..

[76] Harada H., Miyamoto K., Yamashita Y., Nakano K., Taniyama K., Miyata Y., Ohdan H., Okada M. (2013). Methylation of breast cancer susceptibility gene 1 (BRCA1) predicts recurrence in patients with curatively resected stage I non–small cell lung cancer. Cancer.

[77] Gao W., Zhou Z., Liu Y., Liu D., Feng Q., Xu Y. (2016). Prognostic significance of BRCA1 and RASSF1A promoter hypermethylation in non-small cell lung cancer patients. Int. J. Clin. Exp. Pathol..

[78] Nguyen Q.N., Vuong L.D., Truong V.-L., Van Ta T., Nguyen N.T., Nguyen H.P., Chu H.H. (2019). Genetic and epigenetic alterations of the EGFR and mutually independent association with BRCA1, MGMT, and RASSF1A methylations in Vietnamese lung adenocarcinomas. Pathol.-Res. Pract..

[79] Botana-Rial M., De Chiara L., Valverde D., Leiro-Fernández V., Represas-Represas C., del Campo-Pérez V., Fernández-Villar A. (2012). Prognostic value of aberrant hypermethylation in pleural effusion of lung adenocarcinoma. Cancer Biol. Ther..

[80] Wang Y., Zhang D., Zheng W., Luo J., Bai Y., Lu Z. (2008). Multiple gene methylation of nonsmall cell lung cancers evaluated with 3-dimensional microarray. Cancer.

[81] Do H., Wong N.C., Murone C., John T., Solomon B., Mitchell P.L., Dobrovic A. (2014). A critical re-assessment of DNA repair gene promoter methylation in non-small cell lung carcinoma. Sci. Rep..

[82] Safar A.M., Spencer H., Su X., Coffey M., Cooney C.A., Ratnasinghe L.D., Hutchins L.F., Fan C.-Y. (2005). Methylation profiling of archived non–small cell lung cancer: A promising prognostic system. Clin. Cancer Res..

[83] Ter Brugge P., Kristel P., Van Der Burg E., Boon U., De Maaker M., Lips E., Mulder L., De Ruiter J., Moutinho C., Gevensleben H. (2016). Mechanisms of therapy resistance in patient-derived xenograft models of BRCA1-deficient breast cancer. J. Natl. Cancer Inst..

[84] Besselink N., Keijer J., Vermeulen C., Boymans S., de Ridder J., van Hoeck A., Cuppen E., Kuijk E. (2023). The genome-wide mutational consequences of DNA hypomethylation. Sci. Rep..

[85] Suzuki K., Suzuki I., Leodolter A., Alonso S., Horiuchi S., Yamashita K., Perucho M. (2006). Global DNA demethylation in gastrointestinal cancer is age dependent and precedes genomic damage. Cancer Cell.

[86] Varella-Garcia M. (2010). Chromosomal and genomic changes in lung cancer. Cell Adhes. Migr..

[87] Whang-Peng J., Kao-Shan C., Lee E., Bunn P., Carney D., Gazdar A., Minna J. (1982). Specific chromosome defect associated with human small-cell lung cancer: Deletion 3p (14–23). Science.

[88] Balsara B.R., Testa J.R. (2002). Chromosomal imbalances in human lung cancer. Oncogene.

[89] Tseng R.C., Chang J.W., Hsien F.J., Chang Y.H., Hsiao C.F., Chen J.T., Chen C.Y., Jou Y.S., Wang Y.C. (2005). Genomewide loss of heterozygosity and its clinical associations in non small cell lung cancer. Int. J. Cancer.

[90] Aylon Y., Oren M. (2011). p53: Guardian of ploidy. Mol. Oncol..

[91] Tian T., Shan L., Yang W., Zhou X., Shui R. (2019). Evaluation of the BRCAness phenotype and its correlations with clinicopathological features in triple-negative breast cancers. Hum. Pathol..

[92] Tsyganov M.M., Ibragimova M.K., Garbukov E.Y., Bragina O.D., Karchevskaya A.A., Usynin E.A., Litvyakov N.V. (2022). Determination of BRCAness Phenotype in Breast Tumors for the Appointment of Neoadjuvant Chemotherapy Based on Platinum and Taxanes. Int. J. Mol. Sci..

[93] French D., Wilkinson M.R., Yang W., de Chaisemartin L., Cook E.H., Das S., Ratain M.J., Evans W.E., Downing J.R., Pui C.-H. (2005). Global gene expression as a function of germline genetic variation. Hum. Mol. Genet..

[94] Knudson A.G. (2001). Two genetic hits (more or less) to cancer. Nat. Rev. Cancer.

[95] Negrini S., Gorgoulis V.G., Halazonetis T.D. (2010). Genomic instability—An evolving hallmark of cancer. Nat. Rev. Mol. Cell Biol..

[96] Kops G.J., Weaver B.A., Cleveland D.W. (2005). On the road to cancer: Aneuploidy and the mitotic checkpoint. Nat. Rev. Cancer.

[97] Sokolenko A.P., Savonevich E.L., Ivantsov A.O., Raskin G.A., Kuligina E.S., Gorodnova T.V., Preobrazhenskaya E.V., Kleshchov M.A., Tiurin V.I., Mukhina M.S. (2017). Rapid selection of BRCA1-proficient tumor cells during neoadjuvant therapy for ovarian cancer in BRCA1 mutation carriers. Cancer Lett..

[98] Sokolenko A.P., Bizin I.V., Preobrazhenskaya E.V., Gorodnova T.V., Ivantsov A.O., Iyevleva A.G., Savonevich E.L., Kotiv K.B., Kuligina E.S., Imyanitov E.N. (2020). Molecular profiles of BRCA1-associated ovarian cancer treated by platinum-based therapy: Analysis of primary, residual and relapsed tumors. Int. J. Cancer.

[99] Tsyganov M.M., Frolova A.A., Kravtsova E.A., Tsydenova I.A., Ibragimova M.K. (2024). Modulation of homologous recombination gene activity in breast tumor cells in an in vitro model. Uspekhi Mol. Onkol. = Adv. Mol. Oncol..

[100] Min A., Im S.-A., Kim D.K., Song S.-H., Kim H.-J., Lee K.-H., Kim T.-Y., Han S.-W., Oh D.-Y., Kim T.-Y. (2015). Histone deacetylase inhibitor, suberoylanilide hydroxamic acid (SAHA), enhances anti-tumor effects of the poly (ADP-ribose) polymerase (PARP) inhibitor olaparib in triple-negative breast cancer cells. Breast Cancer Res..

[101] Mio C., Gerratana L., Bolis M., Caponnetto F., Zanello A., Barbina M., Di Loreto C., Garattini E., Damante G., Puglisi F. (2019). BET proteins regulate homologous recombination-mediated DNA repair: BRCAness and implications for cancer therapy. Int. J. Cancer.

[102] Medina P.P., Ahrendt S.A., Pollan M., Fernandez P., Sidransky D., Sanchez-Cespedes M. (2003). Screening of homologous recombination gene polymorphisms in lung cancer patients reveals an association of the NBS1-185Gln variant and p53 gene mutations. Cancer Epidemiol. Biomark. Prev..

