# Supplementary material for: BRCA1 Gene as a Potential Marker for Lung Cancer Therapy
Source: Int J Mol Sci. 2026 Jul 17;27(14):6364. doi: 10.3390/ijms27146364 (PMC13410211; doi:10.3390/ijms27146364)
Supplement: Supplementary file 1 [file ijms-27-06364-s001.zip › Figure S1.pdf]

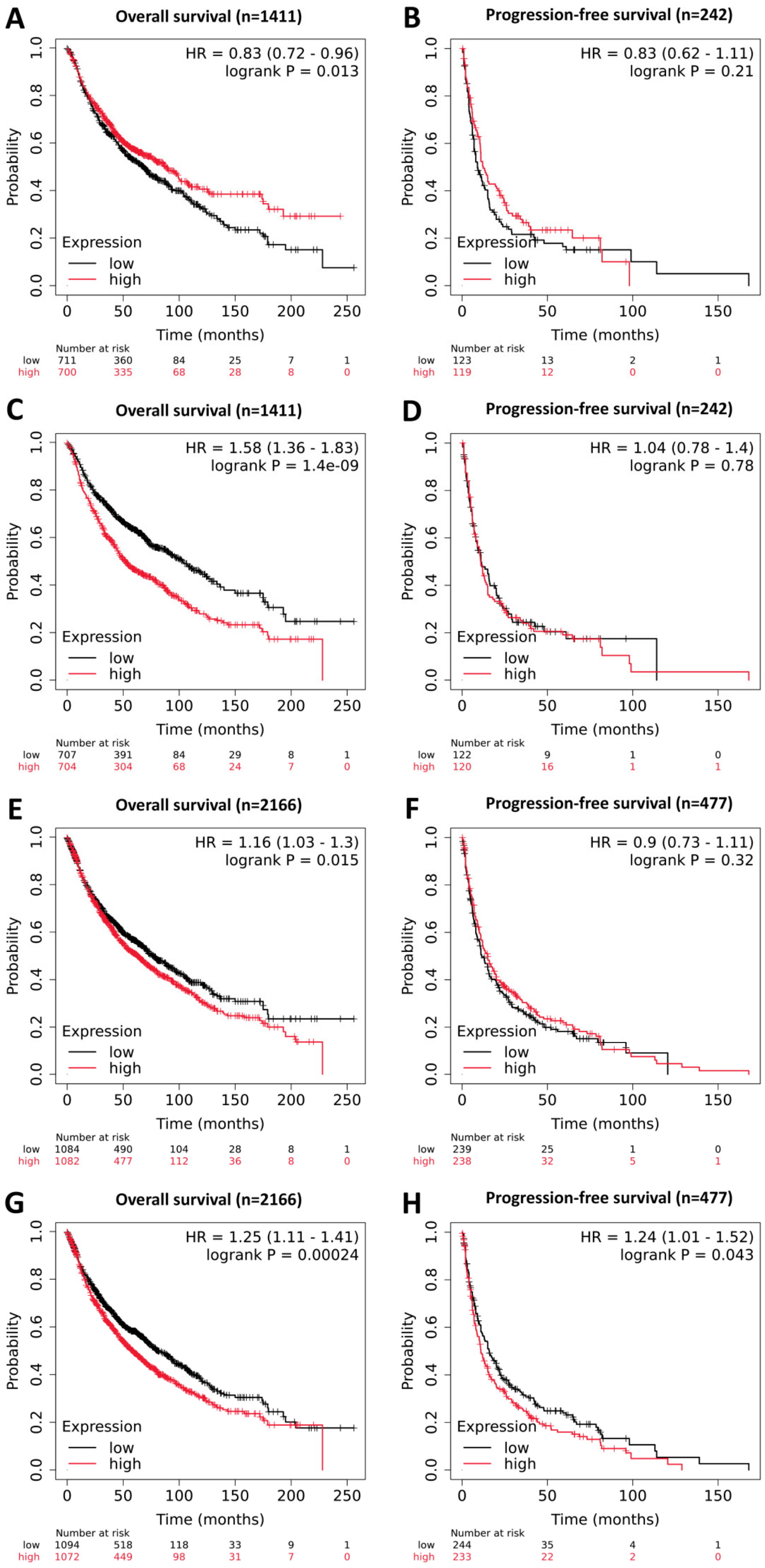
A
Overall survival (n=1411)
HR = 0.83 (0.72 - 0.96)
logrank P = 0.013
Expression
low
high
Probability
Time (months)
Number at risk
low 711 360 84 25 7 1
high 700 335 68 28 8 0
B
Progression-free survival (n=242)
HR = 0.83 (0.62 - 1.11)
logrank P = 0.21
Expression
low
high
Probability
Time (months)
Number at risk
low 123 13 2 1
high 119 12 0 0
C
Overall survival (n=1411)
HR = 1.58 (1.36 - 1.83)
logrank P = 1.4e-09
Expression
low
high
Probability
Time (months)
Number at risk
low 707 391 84 29 8 1
high 704 304 68 24 7 0
D
Progression-free survival (n=242)
HR = 1.04 (0.78 - 1.4)
logrank P = 0.78
Expression
low
high
Probability
Time (months)
Number at risk
low 122 9 1 0
high 120 16 1 1
E
Overall survival (n=2166)
HR = 1.16 (1.03 - 1.3)
logrank P = 0.015
Expression
low
high
Probability
Time (months)
Number at risk
low 1084 490 104 28 8 1
high 1082 477 112 36 8 0
F
Progression-free survival (n=477)
HR = 0.9 (0.73 - 1.11)
logrank P = 0.32
Expression
low
high
Probability
Time (months)
Number at risk
low 239 25 1 0
high 238 32 5 1
G
Overall survival (n=2166)
HR = 1.25 (1.11 - 1.41)
logrank P = 0.00024
Expression
low
high
Probability
Time (months)
Number at risk
low 1094 518 118 33 9 1
high 1072 449 98 31 7 0
H
Progression-free survival (n=477)
HR = 1.24 (1.01 - 1.52)
logrank P = 0.043
Expression
low
high
Probability
Time (months)
Number at risk
low 244 35 4 1
high 233 22 2 0

Figure S1. Overall survival (A, C, E and G) and progression-free survival (B, D, F and H) curves for patients with non-small cell lung cancer, based on the expression levels of the *BARD1* (A and B), *BRIP1* (C and D), *PARP1* (E and F) and *RAD51* (G and H) genes, respectively.
