# Supplementary material for: BRCA1 Gene as a Potential Marker for Lung Cancer Therapy
Source: Int J Mol Sci. 2026 Jul 17;27(14):6364. doi: 10.3390/ijms27146364 (PMC13410211; doi:10.3390/ijms27146364)
Supplement: Supplementary file 1 [file ijms-27-06364-s001.zip › Table S1 full version.pdf]

Table S1. Mutations of *BRCA1* and *BRCA2* genes in lung tumor.

Number of patients	Mutation (Nucleotide change/Protein change)	Reference Sequence	Mutation type	Mutation class	Frequency, abs. n., (%)	References
BRCA1						
-	c.139A>G (p.Cys47Arg) / C47R	rs80357370	Missense	Pathogenic	-	[1]
-	c.2143_2155delinsTCTTT (p.Thr715SerfsTer8) / T668fs	rs1567796807	Frameshift delins	Pathogenic	-	[2]
201	c.122A>T (p.His41Leu) / H41L	rs80357276	Missense	Pathogenic/Likely pathogenic	1 (0.5)	[3]
	c.4035del (p.Glu1346fs) / E1299fs	rs80357711	Frameshift deletion	Pathogenic	1 (0.5)	
	c.520C>T (p.Gln174Ter) / Q174*	rs1567806048	Stop-gain	Pathogenic	1 (0.5)	
1026	- / p.I1824fs	-	Frameshift	Likely pathogenic	1 (0.1)	[4]
	- / IVS5332+1G>-	-	Missense	Likely pathogenic	1 (0.1)	
15	c.5095C>T (p.Arg1699Trp) / R1699W	rs55770810	Missense	Pathogenic	3 (20.0)	[5]
	c.5074G>C (p.Asp1692His) / D1692H	rs80187739	Missense	Pathogenic	4 (26.7)	
	c.5579A>C (p.His1860Pro) / H1860P	rs201196020	Missense	Benign	4 (26.7)	
	c.5576C>G (p.Pro1859Arg) / P1859R	rs80357322	Missense	Benign	11 (73.3)	
	c.5558A>C (p.Tyr1853Ser) / Y1853S	rs80357258	Missense	Benign	4 (26.7)	
	c.5531T>G (p.Leu1844Arg) / L1844R	rs80357323	Missense	Benign	7 (46.7)	
	c.5411T>A (p.Val1804Asp) / V1804D	rs80356920	Stop-gain	Benign	2 (13.3)	
	c.5402G>A (p.Gly1801Asp) / G1801D	rs531210457	Missense	Benign	1 (6.7)	
	c.5347A>T (p.Met1783Leu) / M1783L	-		Benign	13 (86.7)	
	c.5198A>G (p.Asp1733Gly) / D1733G	rs80357270	Missense	Benign	7 (46.7)	
	c.5158A>G (p.Thr1720Ala) / T1720A	rs56195342	Missense	Benign	9 (60.0)	
	c.5044G>A (p.Glu1682Lys) / E1682K	rs80356958	Missense	Benign	11 (73.3)	
	c.5024C>T (p.Thr1675Ile) / T1675I	rs150729791	Missense	Benign	4 (26.7)	
	c.4991T>C (p.Leu1664Pro) / L1664P	rs80357314	Missense	Benign	1 (6.7)	
BRCA2						
109	c.1114A>C (p.Asn372His) / p.(N372H)	rs144848	Missense	Benign	12 (11)	[6]
-	c.9976A>T (p.Lys3326Ter)	rs11571833	Stop-gain	Benign	-	[7]
52	p.Asn235Asp / N235D	-	Missense	Benign	1 (1.9)	[8]
	c.841G>A (p.Asp281Asn) / D281N	rs80359088	Missense	Likely benign	1 (1.9)	

	c.887A>G (p.Tyr296Cys) / Y296C	rs45457795	Missense	VUS	1 (1.9)	
	c.1279G>C (p.Asp427His) / D427H	rs147797288	Missense	VUS	1 (1.9)	
	c.1662T>G (p.Cys554Trp) / C554W	rs80358451	Missense	Benign	1 (1.9)	
	c.2698A>G (p.Asn900Asp) / N900D	rs55736268	Missense	Likely benign	1 (1.9)	
	c.3450dup (p.Ile1151TyrfsTer7) / I1151F	rs397507668	Frameshift duplication	Pathogenic	1 (1.9)	
	c.3458A>G (p.Lys1153Arg) / K1153R	rs80358594	Missense	CIP	1 (1.9)	
	c.3824T>C (p.Ile1275Thr) / I1275T	rs80358625	Missense	CIP	1 (1.9)	
	c.4421A>G (p.Lys1474Arg) / K1474R	rs780660669	Missense	CIP	1 (1.9)	
	c.4482T>G (p.Ser1494Arg) / S1494R	rs761826517	Missense	VUS	1 (1.9)	
	c.4483G>A (p.Val1495Ile) / V1495I	rs80358680	Missense	VUS	1 (1.9)	
	c.4918C>T (p.His1640Tyr) / H1640Y	rs765892492	Missense	VUS	1 (1.9)	
	c.5065G>A (p.Ala1689Thr) / A1689T	rs767200163	Missense	VUS	1 (1.9)	
	c.5414A>G (p.Asn1805Ser) / N1805S	rs80358765	Missense	CIP	1 (1.9)	
	c.5552T>G (p.Ile1851Ser) / I1851S	rs80358776	Missense	Benign	1 (1.9)	
	c.5870T>C (p.Ile1957Thr) / I1957T	rs587782320	Missense	CIP	1 (1.9)	
	c.7095T>A (p.His2365Gln) / H2365Q	rs370708814	Missense	CIP	1 (1.9)	
	c.7226C>T (p.Pro2409Leu) / P2409L	rs759999459	Missense	VUS	1 (1.9)	
	c.8732C>G (p.Ala2911Gly) / A2911G	rs80359130	Missense	VUS	1 (1.9)	
	c.9155G>A (p.Arg3052Gln) / R3052Q	rs80359171	Missense	CIP	1 (1.9)	
	c.9247A>G (p.Lys3083Glu) / K3083E	rs80359190	Stop-gain	VUS	1 (1.9)	
	c.9592T>C (p.Cys3198Arg) / C3198R	rs80359229	Missense	Benign	1 (1.9)	
	c.9905G>A (p.Arg3302Lys) / R3302K	rs80359249	Missense	CIP	1 (1.9)	
	c.9925G>A (p.Glu3309Lys) / E3309K	rs80359251	Missense	CIP	1 (1.9)	
11	c.1813dup (p.Ile605AsnfsTer11) / c.1813del (p.Ile605fs)	rs80359306	Frameshift duplication	Pathogenic	1 (9)	[9]
-	c.8350C > T (p.Arg2784Trp) / R2784W	rs80359075	Missense	Pathogenic/Likely pathogenic	-	[1]
22	c.5171T>C (p.Ile1724Thr)	rs80358743	Missense	CIP	1 (4.5)	[10]
15	c.1114A>C (p.Asn372His) / p.(N372H)	rs144848	Missense	Benign	3 (20)	[11]
	c.2303_2304del / p.(p.Thr768fs)	-	-	-	1 (6.6)	
126	c.9097del (p.Thr3033fs) / c.9097dup (p.Thr3033fs)	rs397507419	Frameshift deletion	Pathogenic	24 (19)	[12]
148	c.8187G>T (p.Lys2729Asn) / K2729N	rs80359065	Missense	Benign	41 (27,7)	[13]

	c.943T>A (p.Cys315Ser) / -	rs79483201	Missense	Benign	26 (17,6)	
	c.6325G>A (p.Val2109Ile) / V2109I	rs79456940	Missense	Benign	12 (8,1)	
	c.6322C>T (p.Arg2108Cys) / R2108C	rs55794205	Missense	Benign	11 (7,4)	
	c.7469T>C (p.Ile2490Thr) / I2490T	rs11571707	Missense	Benign	10 (6,8)	
	c.1744A>C (p.Thr582Pro) / T582P	rs80358457	Missense	Benign	5 (3,4)	
	c.5164_5165del (p.Ser1722fs) / -	rs80359490	Frameshift deletion	Pathogenic	1 (0,7)	
-	c.1688G>A (p.Trp563Ter) / W563*	rs1566224110	Stop-gain	-	1 (9)	[2]
	c.9065G>C (p.Arg3022Thr) / R3022T	-	Missense	VUS	1 (9)	
	c.5082A>G (p.Arg1694=) / R1694R	-	Synonymous	-	1 (9)	
	c.9299T>A (p.Leu3100*) / -	-	Stop-gain	-	1 (9)	
	c.8026A>C (p.Met2676Leu) / M1032L	-	Missense	VUS	1 (9)	
	c.8951C>T (p.Ser2984Leu) / S2984L	rs149341527	Splice region	Likely benign	1 (9)	
	c.6952C>T (p.Arg2318Ter) / R2318*	rs80358920	Stop-gain	Pathogenic	1 (9)	
	c.7718T>G (p.Leu2573Ter) / L2573*	rs786203680	Stop-gain	Pathogenic	1 (9)	
	c.5080A>T (p.Arg1694Ter) / R1694*	rs200265692	Stop-gain	Pathogenic	1 (9)	
	c.7805+1G>A p.? / -	rs81002809	Splice donor	Pathogenic	1 (9)	
	c.8951C>G (p.Ser2984Ter) / S2984*	rs80359146	Stop-gain	Pathogenic	1 (9)	
201	c.1749G>C (p.Leu583Phe) / L1283F	-	-	Not reported / Probably damaging	1 (0.5)	[3]
	c.2974A>T (p.Lys992*) / K992*	-	Stop-gain	Not reported	1 (0.5)	
	c.5593_5594delAT (p.Phe1866TyrfsTer6) / -	-	Frameshift deletion	Pathogenic	1 (0.5)	
	c.5946delT (p.Ser1982ArgfsTer22) / S1982fs	rs80359550	Frameshift deletion	Pathogenic	2 (1)	
	c.6013G>C (p.Asp2005His) / D2005H	-	Missense	Not reported / Probably damaging	1 (0.5)	
	c.7496A>C (p.Q2499P) / c.7495C>G (p.Gln2499Glu)	rs876660447	Missense	Not reported / Probably damaging	1 (0.5)	
	c.7616_7617delAGinsGT (p.Gln2539Arg) / Q2539R	rs144728108	Missense	Not reported	1 (0.5)	
	c.7977_7989delATATGATACGGAA (p.Y2660Lfs*9) / c.7979_7991del (p.Tyr2660fs)	rs730881614	Frameshift deletion	Not reported	1 (0.5)	
	c.9868G>C (p.Ala3290Pro) / A3290P	rs2095950515	Missense	Not reported	1 (0.5)	

	c.9956C>A (p.Ser3319Tyr) / S3287Y	-	Missense	Not reported	1 (0.5)	
1026	c.5163dup (p.Ser1722fs) / p.S1722fs	-	Frameshift duplication	Pathogenic	1 (0.1)	[4]
	- / p.Ile2149fs	-	Frameshift	Pathogenic	1 (0.1)	
	- / p.Lys936fs	-	Frameshift	Pathogenic	1 (0.1)	
	c.1792dup (p.Thr598fs) / p.T598fs	rs886040389	Frameshift duplication	Pathogenic	1 (0.1)	
	c.3109C>T p.(Gln1037Ter) / p.Q1037X	rs80358557	Stop-gain	Pathogenic	1 (0.1)	
	c.3165_3168del (p.Asn1055fs) / p.N1055fs	rs1566227892	Frameshift deletion	Likely pathogenic	1 (0.1)	
63	c.5427C>A (p.Cys1809Ter) / C1809*	rs80359791	Stop-gain	Pathogenic	1 (1,6)	[14]
15	c.71T>G (p.Leu24Ter) / L24*	rs397507902	Stop-gain	Pathogenic	4 (26.7)	[5]
	c.92G>A (p.Trp31Ter) / W31*	rs397508045	Stop-gain	Pathogenic	4 (26.7)	
	c.53G>A (p.Arg18His) / R18H	rs80358762	Missense	Benign	11 (73.3)	
	c.128A>G (p.Asn43Ser) / N43S	rs2072285447	Missense	Benign	13 (86.7)	
	c.167A>C (p.Asn56Thr) / N56T	rs80358454	Missense	Benign	1 (6.7)	
	c.223G>C (p.Ala75Pro) / A75P	rs28897701	Missense	Benign	1 (6.7)	
	c.322A>C (p.Asn108His) / N108H	rs80358567	Missense	Benign	5 (33.3)	
	c.502C>A (p.Pro168Thr) / P168T	rs80358726	Missense	Benign	5 (33.3)	
	c.865A>C (p.Asn289His) / N289H	rs766173	Missense	Benign	2 (13.3)	
	c.868G>C (p.Val290Leu) / V290L	rs1555281616	Missense	Likely Benign	1 (6.7)	

Note: Pathogenic – pathogenic mutation; CIP – Conflicting interpretations of pathogenicity; VUS - Variants of uncertain significance.

References

1. Donner, I.; Katainen, R.; Sipilä, L.J.; Aavikko, M.; Pukkala, E.; Aaltonen, L.A. Germline mutations in young non-smoking women with lung adenocarcinoma. *Lung Cancer* **2018**, *122*, 76-82.
2. Hu, X.; Yang, D.; Li, Y.; Li, L.; Wang, Y.; Chen, P.; Xu, S.; Pu, X.; Zhu, W.; Deng, P.; et al. Prevalence and clinical significance of pathogenic germline BRCA1/2 mutations in Chinese non-small cell lung cancer patients. *Cancer biology & medicine* **2019**, *16*, 556.
3. Ricciuti, B.; Recondo, G.; Spurr, L.F.; Li, Y.Y.; Lamberti, G.; Venkatraman, D.; Umeton, R.; Cherniack, A.D.; Nishino, M.; Sholl, L.M.; et al. Impact of DNA damage response and repair (DDR) gene mutations on efficacy of PD-(L) 1 immune checkpoint inhibition in non–small cell lung cancer. *Clinical Cancer Research* **2020**, *26*, 4135-4142.
4. Liu, M.; Liu, X.; Suo, P.; Gong, Y.; Qu, B.; Peng, X.; Xiao, W.; Li, Y.; Chen, Y.; Zeng, Z.; et al. The contribution of hereditary cancer-related germline mutations to lung cancer susceptibility. *Translational Lung Cancer Research* **2020**, *9*, 646.
5. Wang, L.; Ma, Y.; Han, W.; Yang, Q.; Jamil, M. Whole exome sequencing reveals clinically important pathogenic mutations in DNA repair genes across lung cancer patients. *American journal of cancer research* **2023**, *13*, 4989.
6. Medina, P.P.; Ahrendt, S.A.; Pollan, M.; Fernandez, P.; Sidransky, D.; Sanchez-Cespedes, M. Screening of homologous recombination gene polymorphisms in lung cancer patients reveals an association of the NBS1-185Gln variant and p53 gene mutations. *Cancer Epidemiology Biomarkers & Prevention* **2003**, *12*, 699-704.
7. Wang, Y.; McKay, J.D.; Rafnar, T.; Wang, Z.; Timofeeva, M.N.; Broderick, P.; Zong, X.; Laplana, M.; Wei, Y.; Han, Y.; et al. Rare variants of large effect in BRCA2 and CHEK2 affect risk of lung cancer. *Nature genetics* **2014**, *46*, 736-741.
8. Parry, E.M.; Gable, D.L.; Stanley, S.E.; Khalil, S.E.; Antonescu, V.; Florea, L.; Armanios, M. Germline mutations in DNA repair genes in lung adenocarcinoma. *Journal of Thoracic Oncology* **2017**, *12*, 1673-1678.
9. Fan, Y.; Zhu, X.; Xu, Y.; Lu, X.; Xu, Y.; Wang, M.; Xu, H.; Ding, J.; Ye, X.; Fang, L.; et al. Cell-cycle and DNA-damage response pathway is involved in leptomeningeal metastasis of non–small cell lung cancer. *Clinical Cancer Research* **2018**, *24*, 209-216.
10. Pietanza, M.C.; Waqar, S.N.; Krug, L.M.; Dowlati, A.; Hann, C.L.; Chiappori, A.; Owonikoko, T.K.; Woo, K.M.; Cardnell, R.J.; Fujimoto, J.; et al. Randomized, double-blind, phase II study of temozolomide in combination with either veliparib or placebo in patients with relapsed-sensitive or refractory small-cell lung cancer. *Journal of Clinical Oncology* **2018**, *36*, 2386-2394.
11. Lu, S.; Yu, Y.; Li, Z.; Yu, R.; Wu, X.; Bao, H.; Ding, Y.; Shao, Y.W.; Jian, H. EGFR and ERBB2 germline mutations in Chinese lung cancer patients and their roles in genetic susceptibility to cancer. *Journal of Thoracic Oncology* **2019**, *14*, 732-736.
12. Lu, J.; Zhong, H.; Wu, J.; Chu, T.; Zhang, L.; Li, H.; Wang, Q.; Li, R.; Zhao, Y.; Gu, A.; et al. Circulating DNA-based sequencing guided anlotinib therapy in non-small cell lung cancer. *Advanced Science* **2019**, *6*, 1900721.
13. Fang, W.; Cai, X.; Zhou, H.; Wang, Y.; Zhang, Y.; Hong, S.; Shao, Y.; Zhang, L. BRCA1/2 germline mutations and response to PARP inhibitor treatment in lung cancer. **2019**.
14. Wu, S.; Zhang, Y.; Zhang, Y.; Chen, L.h.; Ouyang, H.f.; Xu, X.; Du, Y.; Ti, X.y. Mutational landscape of homologous recombination-related genes in small-cell lung cancer. *Cancer medicine* **2023**, *12*, 4486-4495.
